# Progress in the Design and Application of Chemical and Biological Sensors Based on Atom Transfer Radical Polymerization

**DOI:** 10.3390/bios15110752

**Published:** 2025-11-10

**Authors:** Ning Xia, Fengli Gao, Zhaojiang Yu, Shuaibing Yu, Xinyao Yi

**Affiliations:** 1 Henan Province Key Laboratory of New Opto-Electronic Functional Materials, College of Chemistry and Chemical Engineering, Anyang Normal University, Anyang 455000, China; flgao@aynu.edu.cn (F.G.); yzj86jiang@aynu.edu.cn (Z.Y.); 2 College of Chemical and Environmental Engineering, Anyang Institute of Technology, Anyang 455000, China; bingsy96@njust.edu.cn; 3 College of Chemistry and Chemical Engineering, Central South University, Changsha 410083, China

**Keywords:** atom transfer radical polymerization, chemical sensors, biosensors, polymers, signal amplification

## Abstract

Atom transfer radical polymerization (ATRP) is a leading reversible deactivation radical polymerization method. It has become an emerging technology to synthesize well-defined, tailor-made polymers, promoting the development of advanced materials (e.g., bioconjugates and nanocomposites) with precisely designed and controlled macromolecular architectures. ATRP-produced polymers or polymeric materials have been successfully applied in the fields of drug delivery, tissue engineering, sample separation, environmental monitoring, bioimaging, clinical diagnostics, etc. In this review, we systematically summarize the progress of ATRP-based chemical and biological sensors in different application fields, including ion sensing, small-molecule detection, bioimaging, and signal amplification for biosensors. Finally, we briefly outline the prospects and future directions of ATRP. This review is expected to provide a fundamental and timely understanding of ATRP-based sensors and guide the design of novel materials and methods for sensing applications.

## 1. Introduction

Atom transfer radical polymerization (ATRP) is a landmark technology in the field of controlled radical polymerization. For the pioneering development of ATRP in 1995, Matyjaszewski’s group first reported copper-based ATRP [1], while Sawamoto’s group independently developed ruthenium-based ATRP at the same time [2]. As a pivotal polymerization technique, ATRP has opened up a novel avenue for the design and synthesis of functional materials. The core advantage of ATRP lies in its capability to precisely regulate the molecular structures of polymers, serving as the fundamental basis for its extensive application in the field of material synthesis. In comparison with conventional free-radical polymerization, ATRP can establish a dynamic equilibrium between active and dormant species via reversible atom transfer reactions between transition-metal catalysts and alkyl halide initiators [3]. This not only enables efficient control over the molecular weight distribution of polymers but also facilitates the precise fabrication of complex topological architectures, including block, graft, and star configurations [4,5]. Such a unique capability has endowed ATRP with irreplaceable application values in different fields. For instance, ATRP-produced degradable polymers have been used to deliver peptide, protein, and antibody-based drugs [6,7], which are termed as polymer therapeutics. Various polymers and polymeric materials prepared by ATRP reactions have been applied for the separation of biomolecules and bacteria [8,9,10,11,12,13]. ATRP techniques have been employed to modify the sensing surface for efficient immobilization of recognition elements with low non-specific adsorption [14,15,16,17,18,19,20,21,22,23,24,25].

A sensor is a core device used for information acquisition. Its performance is highly dependent on the synergistic interaction between the recognition unit and signal transduction interface. In the fabrication of conventional sensors, the immobilization of recognition molecules often suffers from issues such as low loading capacity, uneven distribution, and poor stability, which may hinder the breakthrough in detection sensitivity and selectivity [26]. In contrast, ATRP technologies can enable the direct grafting of functional polymer chains onto the surface of electrodes, nanomaterials, or biochips, achieving high-density and ordered modification of recognition elements [27]. For instance, in electrochemical sensors, grafting the polymer chains with specific chelating groups onto the electrode surface via surface-initiated ATRP (SI-ATRP) can significantly enhance the capture efficiency of heavy-metal ions and lower the limit of detection down to the ppb or even ppt level [28]. Its advantage stems from the unique surface-confined polymerization mechanism. Unlike solution ATRP, which requires post-synthesis immobilization to yield soluble polymer chains, SI-ATRP can directly anchor the initiators on the solid surface, enabling in situ growth of dense, uniformly distributed polymer brushes. In optical sensors, fluorescence-labeled polymers synthesized via ATRP can enable highly selective fluorescent responses toward small biological molecules [29]. In addition, the controllability of ATRP can facilitate the multifunctional integration of sensors. Through the sequential polymerization of different monomers, polymer brushes with multiple responsive units can be constructed on the surface of a single substrate, enabling the simultaneous detection of multiple targets in complex samples [30,31]. Furthermore, ATRP reactions exhibit high compatibility with the polymerization conditions, laying a foundation for in vivo sensing and in situ detection. In recent years, with the development of advanced ATRP techniques, such as activators generated by electron transfer ATRP (AGET ATRP) [32], electrochemically mediated ATRP (eATRP) [33,34], enzyme-catalyzed ATRP [35], and photo-induced ATRP (photo-ATRP) [36], the sensitivity of ATRP systems toward air and water has been significantly reduced, drastically decreasing the required dosage of catalysts. This effectively addresses the issues of biotoxicity and signal interference caused by catalyst residues in conventional ATRP methods, thereby breaking through the application bottlenecks of ATRP in the sensing fields. Currently, ATRP-based chemical and biological sensors have been widely developed and have shown great potential in the fields of environmental monitoring, food safety, clinical diagnosis, etc.

ATRP has sparked great research enthusiasm worldwide since its first report in 1995. Some interesting review papers have been reported to address the applications, current status, and future challenges of ATRP [37,38,39,40,41,42,43,44]. For example, Matyjaszewski and co-workers have summarized the current status and outlook for ATRP [37,38,41]. Yazdi et al. outlined the progress in ATRP-derived functional materials for biomedical applications [39]. Although limited chapters have involved reversible addition–fragmentation chain-transfer (RAFT)-based sensors in early reviews [40,42,43,44], there is no systematic review addressing the design principle and sensing application of ATRP. Given the unique advantages of ATRP in structural fabrication and its rapid development in the sensing fields, this review systematically summarizes the progress of ATRP technology in the design and application of chemical and biological sensors, including ion sensing, small-molecule detection, bioimaging, and signal amplification for biosensors. In addition, the merits and inherent limitations of ATRP methods across diverse sensing platforms and their future development trajectories are outlined. The overarching goal of this review is to provide theoretical frameworks and technical perspectives that will inform the development of novel polymers and methods for sensing applications.

## 2. Mechanisms and Types of ATRP Techniques

**Scheme 1:**
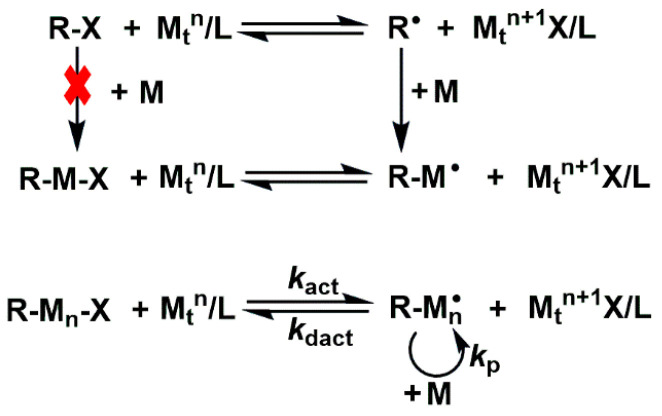
The reaction mechanism of classical ATRP.

As an important type of reversible-deactivation radical polymerization (RDRP) technique, the mechanism of classical ATRP is illustrated in Scheme 1. Herein, X represents the halogen atom (such as Cl and Br) or SCN; M_t_^n^, L, and M denote the transition-metal catalyst, ligand, and monomer, respectively. K_p_ is the rate constant of the polymerization reaction, K_act_ is the rate constant of the activation reaction, and K_dact_ is the rate constant of the deactivation reaction. In the ATRP reaction system, the reaction first occurs between the halogen-containing initiator (R−X) and the metal complex (M_t_^n^/L). During the initiation stage, the low-valent metal complex M_t_^n^/L abstracts X from R−X, generating a radical R• and a high-valent metal halide M_t_^n+1^−X/L. The R• radical exhibits high reactivity and can attack the carbon–carbon double bond in M, thereby forming a chain radical R−M•. The newly formed chain radical R−M• shows strong reactivity, which can continue to react with surrounding M monomers. As the reaction proceeds, R−M• continuously induces the polymerization of M, gradually forming a chain-growth radical R−M_n_•. Subsequently, the highly active R−M_n_• can undergo a deactivation reaction with the oxidized M_t_^n+1^−X/L, capturing X therefrom to form a relatively stable dormant species R−M−X. Meanwhile, during the deactivation reaction, the metal ion is reduced from a high-valent state to a low-valent state (M_t_^n+1^ is reduced to M_t_^n^), providing conditions for the activation of R−X in the next cycle. This dynamic equilibrium activation–deactivation process enables the entire polymerization reaction to proceed under relatively mild conditions and can effectively control the molecular weight and distribution of polymers, thereby allowing for the synthesis of polymers with specific structures and properties.

ATRP exhibits prominent advantages in sensor construction. It can precisely regulate the structure, molecular weight, and functional group density of polymers and form uniform and stable sensing coatings with strong compatibility via the SI-ATRP technique. This enables it to be combined with various detection modes to achieve the integration of “recognition–signal amplification”. For this purpose, different types of ATRP techniques have focused on application in the sensing field. As a fundamental method, classical ATRP is often used to construct high-precision sensing interfaces due to its excellent controllability, but its relatively high catalyst loading may introduce metal residue interference, making it suitable for chemical sensors with low purity requirements. Activator regenerated by electron transfer (ARGET) ATRP can continuously regenerate catalysts through reducing agents, reducing the catalyst concentration to 100–1000 ppm. This can minimize metal contamination to the greatest extent possible and make it more suitable for the design of biosensors. In addition, its oxygen tolerance greatly simplifies the operational process. However, AGET ATRP requires the addition of a reducing agent at the initial stage of the reaction to generate an active catalyst, avoiding potential reaction between the reducing agent and monomer. This method is more applicable for the construction of fluorescent sensors containing sensitive functional monomers such as fluorescent monomers. Photo-ATRP can regulate the polymerization process through light irradiation, featuring a high spatiotemporal resolution. This enables precise local modification of sensors or the design of responsive coatings (e.g., light-controlled release of signal molecules) and shows significant potential in the field of intelligent sensing. The eATRP technique can precisely regulate the redox state of catalysts via electrochemical means without the requirement of additional reducing agents. It operates under mild and highly controllable reaction conditions, allowing real-time adjustment of polymerization rate. This makes it particularly suitable for constructing fast-response electrochemical sensors and endows it with unique advantages in the field of on-site rapid detection.

## 3. Polymeric Materials Synthesized by ATRP Techniques for Sensing Applications

### 3.1. Ion Sensing

**Figure 1:**
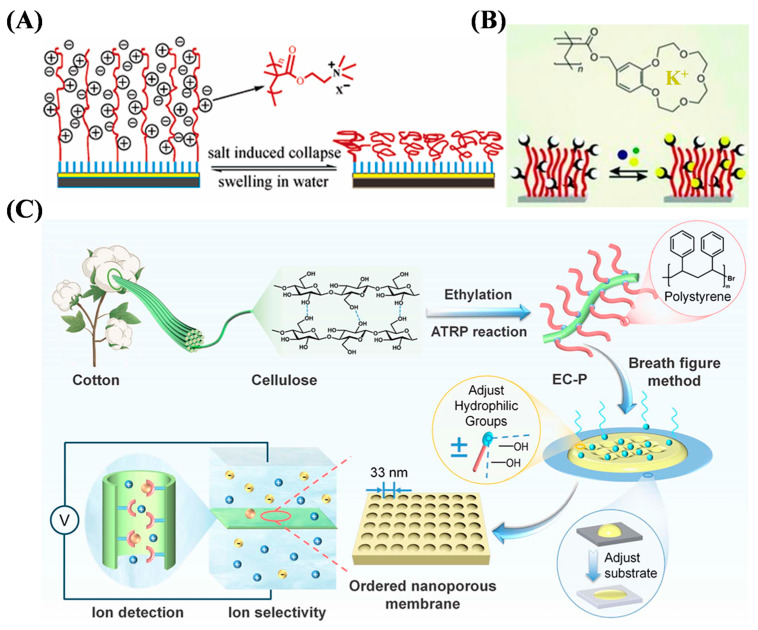
(**A**) Schematic illustration of reversible conformational change of surface-tethered cationic polyelectrolyte brush [45]. Copyright 2007 American Chemical Society. (**B**) Schematic illustration for synthesis of benzo-15-crown-5-functionalized polymer brush via SI-ATRP [46]. Copyright 2010 Wiley-VCH. (**C**) Schematic illustration of ordered nanoporous cellulose-based membrane for ion detection [50]. Copyright 2025 Royal Society of Chemistry.

Ions exist throughout our environment, from biological systems to agriculture and other fields. Important biological processes and mechanisms can be driven by the presence and concentration change of anions and cations, highlighting the importance of ion sensing. The incorporation of biopolymers and conductive polymer materials through various methods has shown great potential for sensitivity and selectivity toward heavy-metal ions. By adopting a dynamic balance between dormant and active species, ATRP allows for the synthesis of well-defined polymers with customized molecular weight distributions, low dispersity, and various architectures. ATRP-based stimuli-responsive smart polymers and polymeric materials have been designed and used for electronic and optical sensing of ions. For example, methacrylate, acrylamide, and crown-based polymer brushes, cellulose-based membranes, and polyacrylonitrile-grafted graphene oxide (GO) composites have been used as the electrode modifiers for sensing of metal ions (Table 1) [45,46,47,48,49,50]. Typically, Zhou et al. suggested that poly[(dimethylamino)ethyl methacrylate] (PDMAEMA) could be grown on a gold surface by SI-ATRP and then quaternized with methane iodide to yield cationic brushes (Q-PDMAEMA) (Figure 1A) [45]. The Q-PDMAEMA brushes in the swollen state showed good permeability for electroactive probes. Some salts and hydrophobic anions could resist electron transport by collapsing the brushes, owing to charge screening and solubility change, which was monitored by electrochemical impedance spectroscopy. Schüwer et al. reported a benzo-15-crown-5-functionalized polymer brush for K^+^ sensing on a SiO_2_ substrate (Figure 1B) [46]. The ATRP initiator (a chlorosilane derivative) was coated on the silicon surface for SI-ATRP of methacryloyl-4′-oxymethylbenzo-15-crown-5. The polymer brush was able to selectively determine K^+^ ions, even in the presence of other metal ions such as Na^+^ and Ca^2+^. The K^+^-induced frequency shift was determined by a quartz crystal microbalance with dissipation measurements. In addition, Hu et al. prepared ordered nanoporous membranes by grafting ethyl cellulose on polystyrene through ethylation and ATRP reactions (Figure 1C) [50]. The nanoporous membranes exhibited an average minimum pore size down to 33 nm. After modification with bovine serum albumin, the membranes could be applied for highly sensitive determination of Cu^2+^ by monitoring the changes in current and conductance.

**Figure 2:**
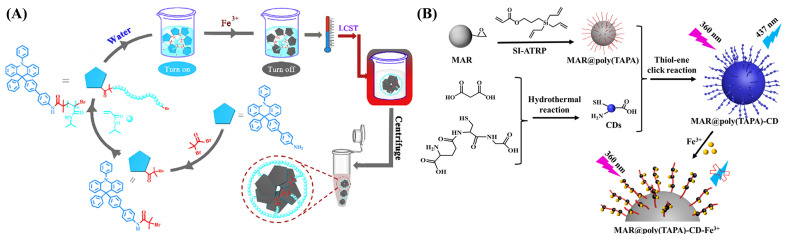
(**A**) Schematic illustration for the synthesis of DPDHR-PNIPAM for Fe^3+^ sensing [51]. Copyright 2020 Elsevier. (**B**) Schematic illustration of Fe^3+^ sensing with bi-functional MAR@poly(TAPA)-CD based on fluorescence quenching [52]. Copyright 2023 Elsevier.

The analyte-induced small disturbance can cause a significant signal change in the fluorescent copolymer with a linear structure in both the single molecular chain and the entire polymer system. Thus, fluorescent polymers exhibit the ability to determine analytes at ultra-low concentrations with a sensitivity typically higher than that of conventional small-molecule probes. Fluorescent copolymers such as PSaAEMA-co-PMPC, hydrazone-based polyvinylpyrrolidone (PVP-NDHIP), and carbon dot (CD)-grafted macroporous adsorption resin (MAR) have been synthesized by ATRP techniques and used for sensing of different ions based on the quenching mechanism [31,51,52,53,54,55]. For example, Cui et al. reported a fluorescent probe for Fe^3+^ sensing using 4′-(9,10-diphenyl-9,10-dihydropyridine-9-yl)-[1,1′-biphenyl]-4-amine (DPDHR-NH_2_) to end poly(N-isopropylacrylamide) (DPDHR-PNIPAM) (Figure 2A) [51]. 2-bromoisobutyryl bromide was reacted with DPDHR-NH_2_ to form an initiator for ATRP of N-isopropylacrylamide monomer. The telechelic polymer is thermosensitive and shows high water solubility at room temperature. It could be used to detect Fe^3+^ at a concentration down to 1.32 μM. At temperatures higher than the lower critical solution temperature (LCST), the polymer chain would collapse to form an aggregate due to the change in hydrophobicity, making the probe easy to separate from water for recyclable applications. In addition, Wang et al. reported a CD-labeled polymeric macroporous adsorption resin (MAR) for the detection and removal of Fe^3+^ (Figure 2B) [52]. The MAR substrate was grafted with 3-(triallylsilyl)propyl acrylate (TAPA) by SI-ATRP, and then CD, synthesized with malonic acid and glutathione as the precursors, was attached onto the polymer surface. The MAR@poly(TAPA)-CD showed blue fluorescence and exhibited a high adsorption ability and quenching efficiency (46.2%) for Fe^3+^.

**Figure 3:**
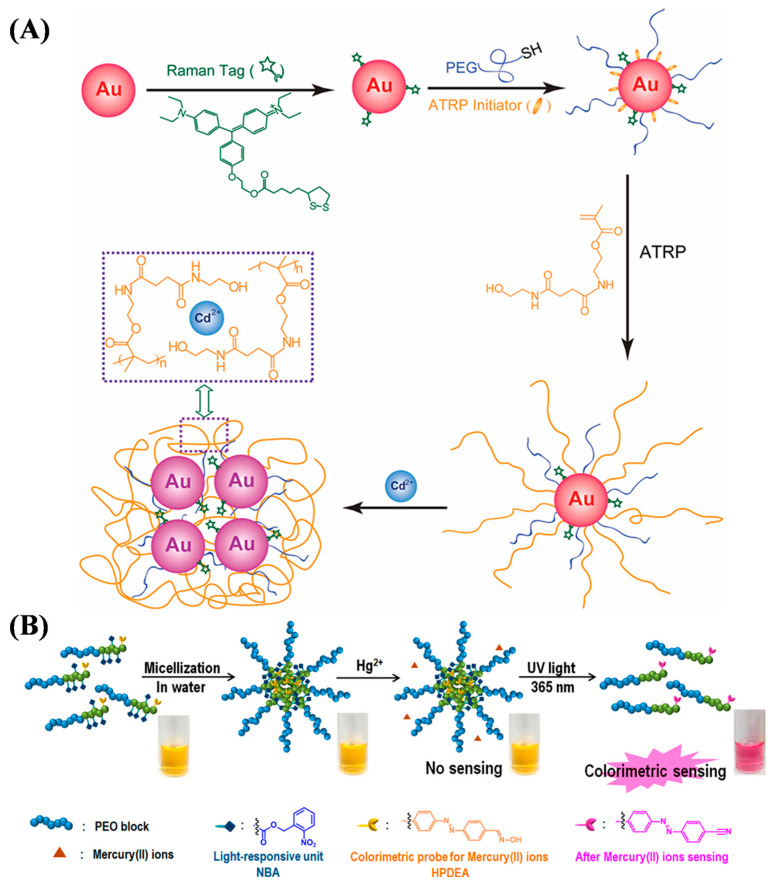
(**A**) Schematic illustration for the fabrication of SERS dye-encoded AuNPs through ligand exchange and SI-ATRP and the working mechanism for selective Cd^2+^ recognition and binding [56]. Copyright 2011 American Chemical Society. (**B**) Schematic diagram of amphiphilic block copolymer with photocleavable nitrobenzyl moiety and oxime group attached to the azo chromophore as a receptor to detect Hg^2+^ [57]. Copyright 2018 American Chemical Society.

In addition to fluorescence methods, other optical methods, such as surface-enhanced Raman spectroscopy (SERS), and colorimetric methods have been developed and used to prepare ATRP-based materials for determining metal ions, such as thermo-responsive and light-responsive double-hydrophilic block copolymer (DHBC), dual-responsive spiropyran-ended poly(N-vinyl caprolactam) (SP PNVCL), cotton fiber grafted with poly(3-sulfopropyl methacrylate potassium salt) and modified with 5,10,15,20-tetrakis(1-methy-4-pyridinio)porphyrin tetra(p-toluenesulfonate) (cotton-PSMP-TMPyP), rhodamine derivative-modified cellulose filter paper, and ring arrays [56,57,58,59,60,61]. SERS can overcome the low sensitivity associated with Raman spectroscopy and benefit the detection of various analytes in combination with localized surface plasmon resonance (LSPR) of plasma nanoparticles. Yin et al. reported a visual SERS sensor based on Cd^2+^-induced aggregation of gold nanoparticles (AuNPs) (Figure 3A) [56]. The AuNPs were encoded with Raman-active dyes and Cd^2+^-chelating polymer brushes by SI-ATRP. Cd^2+^ ions could induce the aggregation of AuNPs by binding to the polymer brushes, turning on the SERS signal with up to 90-fold enhancement with the change of the solution color from red to blue.

Colorimetric sensors involving the color change of indicators induced by analytes can be easily observed with the naked eye or an optical device. Kim et al. reported a polymeric micelle for phototunable sensing of Hg^2+^ (Figure 3B) [57]. The light-responsive block copolymer was prepared by using 2-nitrobenzyl acrylate (NBA) and (*E*)-2-((4-((4-formylphenyl)diazenyl)phenyl)(methyl)amino) ethyl acrylate (FPDEA) as the ATRP monomers and poly(ethylene oxide) (PEO) as the macroinitiator. The aldehyde groups in the polymer were converted into aldoxime groups after reaction with hydroxylamine. The resulting oxime-containing polymer probes could self-assemble into spherical micelles in water. In this method, the PEO block contained a hydrophilic shell and a copolymer of light-responsive NBA, and the Hg^2+^-recognizing HPDEA block formed a hydrophobic core. Hg^2+^ could not approach the oxime unit located in the hydrophobic core. Under UV light irradiation, the photolabile 2-nitrobenzyl moiety was cleaved to transform hydrophobic PNBA into hydrophilic poly(acrylic acid) (PAA). The oxime unit was exposed after the photoinduced dissociation of the micelle into a unimer, allowing for the reaction with Hg^2+^ to form nitrile and the turn-on phototunable sensing. In addition, Lee et al. reported a thermo-responsive double-hydrophilic block copolymer for the colorimetric detection of Hg^2+^ through a temperature-mediated morphological change between a micelle and a unimer [58]. The oxime-containing copolymer was prepared by ATRP of 2-(dimethylamino)ethyl methacrylate (DMAEMA) and FPDEA monomers using PEO macroinitiator and hydroxylamine reagent.

**Table 1:** Overview of ATRP-based materials for ion sensing.

Method	Material	Ion	Linear Range	Detection Limit	Ref.
QCM	Benzo-15-crown-5 polymer	K^+^	0.25–5 mM	–	[46]
DPASV	Polyacrylamide	Pb^2+^	3 × 10^−3^–2000 ng/mL	0.37 μg/mL	[47]
DPASV	PAM/PMAA	Pb^2+^	10^−8^–0.1 mM	2.5 pM	[48]
DPASV	PAN-*g*-GO	Hg(II)	1 × 10^−4^–2 μM	0.06 nM	[49]
I–V	EC-P	Cu^2+^	10^−7^–0.1 nM	0.1 fM	[50]
Fluorescence	DPDHR-PNIPAM	Fe^3+^	0–0.65 μM	1.32 μM	[51]
Fluorescence	MAR@poly(TAPA)-CD	Fe^3+^	10–80 nM	9.74 nM	[52]
Fluorescence	PSaAEMA-*co*-PMPC	Zn^2+^	0–14 mM	–	[53]
Fluorescence	PVP-NDHIP	Al^3+^	–	3.9 nM	[31]
Fluorescence	Cellulose-*g*-PPFMA	Hg^2+^	0–10 mM	0.5 μM	[55]
SERS	AuNPs	Cd^2+^	1–25 μM	1 μM	[56]
Color	PEO_113_-*b*-[p(NBA_10_-*co*-FPDEA_3_)]	Hg(II)	1–10 mM	0.2 mM	[57]
Color	p(DMAEMA-*co*-HPDEA)	Hg(II)	4 × 10^−2^–0.44 mM	0.03 mM	[58]
Color	SP PNVCL	Fe^2+^	1.7 × 10^−2^–0.333 mM	2.98 μM	[59]
Color	Cotton-PSMP-TMPyP	Cd^2+^	0.2–2 mM	0.2 mM	[60]
LSPR	VCHR	Cu^2+^	25–400 pg/mL	25 pg/mL	[61]

Abbreviation: QCM, quartz crystal microbalance; DPASV, differential pulse anodic stripping voltammetry; PAM/PMAA, polyacrylamide-*b*-poly(methacrylic acid); PAN-*g*-GO, polyacrylonitrile-grafted graphene oxide; EC-P, cellulose-grafted polystyrene; DPDHR, 4′-(9,10-diphenyl-9,10-dihydropyridine-9-yl)-[1,1′-biphenyl]-4-amine (DPDHR-NH_2_); PNIPAM, poly(N-isopropylacrylamide; MAR, macroporous adsorption resin of poly(glycidyl methacrylate-*co*-ethylene dimethacrylate); TAPA, 3-(triallylsilyl)propyl acrylate; CD, carbon dot; PSaAEMA-*co*-PMPC, p(2-salicylaldehyde-aminoethyl ethanolamine methacrylate)-*co*-P(2-methacryloyloxyethyl phosphorylcholine); PVP-NDHIP, hydrazone-based polyvinylpyrrolidone; cellulose-*g*-PPFMA, poly(pentafluorophenyl methacrylate)-grafted filter paper; SERS, surface-enhanced Raman scattering; PEO_113_-*b*-[p(NBA_10_-co-FPDEA_3_), block copolymer prepared with 2-nitrobenzyl acrylate and (E)-2-((4-((4-formylphenyl)diazenyl)phenyl)(methyl)amino)ethyl acrylate from poly(ethyleneoxide); DMAEMA, (dimethylamino)ethyl methacrylate; PNBAEMA-*co*-PMPC, polymer prepared with N-Boc-aminoethyl methacrylate and 2-methacryloyloxy ethyl phosphorylcholine; SP-PNVCL, spiropyran-ended poly(N-vinyl caprolactam); cotton-PSMP-TMPyP, cotton fiber grafted with poly(3-sulfopropyl methacrylate potassium salt) and immobilized with 5,10,15,20-tetrakis(1-methy-4-pyridinio)porphyrin tetra(p-toluenesulfonate); VCHR, polyvinyltetrazole–copper hybrid ring; LSPR, localized surface plasmon resonance.

### 3.2. Sensing of Small Molecules

#### 3.2.1. Electrochemical Sensing

**Figure 4:**
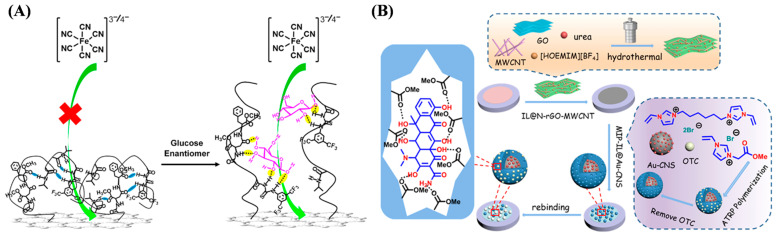
(**A**) Working principle of the electrochemical chiral sensing of monosaccharide enantiomer based on the stimuli-responsive polymer/graphene hybrid-modified screen-printed carbon electrode [68]. Copyright 2016 American Chemical Society. (**B**) Schematic diagram of oxytetracycline detection using surface molecularly imprinted polymer based on ionic liquid and ATRP [73]. Copyright 2022 Elsevier.

Determining the levels of small molecules such as metabolites, neurotransmitters, and hormones can provide useful biological information for diagnosing specific diseases, predicting therapeutic effects, and monitoring health status. Among various sensing technologies, electrochemical sensors have the advantages in point-of-care applications due to their ease of miniaturization, low cost, and fast response. Electroactive small molecules can be easily determined via direct redox reactions on an electrode surface. However, electrochemically inert small molecules can only be quantified by monitoring the electroactive products produced from enzymatic reactions or affinity-induced signal changes by binding to biological receptors or biomimetic receptors on the electrode surface. ATRP-based polymers and polymeric materials have been developed to direct or enzymatical sensing of different small molecules (Table 2), including poly(4-vinylphenylboronic acid) (P(4-VBA))-coated Fe_3_O_4_-modified graphene oxide [62], acryloyloxy ferrocene carboxylate-grafted multiwalled carbon nanotubes (MWCNTs) [63], poly(N-isopropylacrylamide)-coated SiO_2_ core–shell microspheres [64], glucose oxidase-loaded ATRP polymers [65], superoxide dismutase-imprinted poly(ionic liquid) [66], and erythromycin (ERY)-imprinted polymers [67]. In addition, Ding et al. reported an electrochemical method for sensing enantiomers using a stimuli-responsive copolymer/graphene hybrid-modified electrode (Figure 4A) [68]. The stimuli-responsive copolymer consisted of methyl esterified β-Asp-Phe dipeptide (chiral recognition center unit), bis(trifluoromethyl)-modified phenyl thiourea (mediating unit), and poly(N-isopropylacrylamide) (functional switching unit). The three units were grafted onto the reduced graphene oxide (rGO) surface through ATRP. The slight conformational change of the copolymer induced by the weak chiral interaction could greatly facilitate the diffusion of the electroactive probe and monosaccharide onto the electrode interface. The sensor could detect monosaccharide enantiomers at a concentration down to 1 nM. Finally, glucose enantiomers in live cells were discriminated, and the transport mechanisms were investigated by this method.

Molecularly imprinted polymers (MIPs) prepared by imprinting techniques are powerful molecular recognition elements capable of simulating natural recognition entities such as antibodies and biological receptors. They can be used as the acceptors for separating and analyzing complex samples, such as biological fluids and environmental samples [69,70,71,72]. Chen et al. reported an electrochemical sensor for oxytetracycline (OTC) detection by using imprinted AuNPs/carbon nanosphere (Au-CNS) composites (Figure 4B) [73]. The OTC-imprinted MIPs were formed on the surface of Au-CNS supporters through ATRP with ionic liquids (ILs) as functional monomers and cross-linking agents. OTC in the concentration range of 0.02–20 μM has been determined by a sensor electrode coated with IL-modified nitrogen-doped graphene and MWCNT nanocomposites (IL@N-rGO-MWCNT) and MIP-IL@Au-CNS.

**Figure 5:**
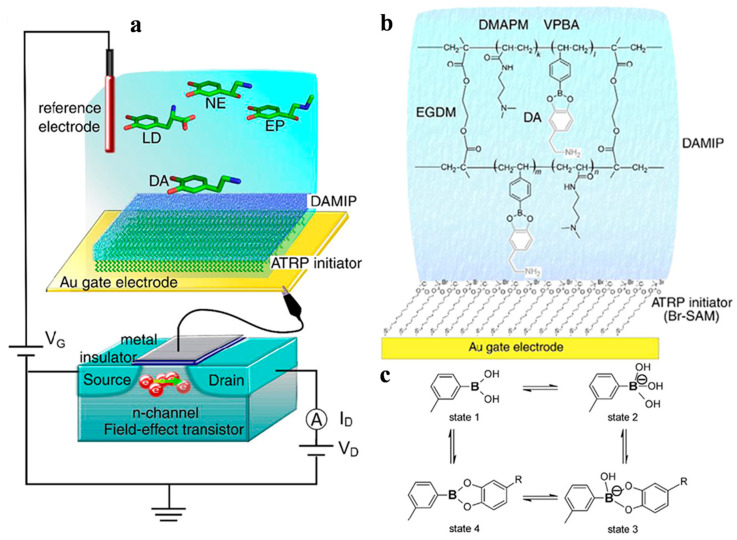
(**a**) Schematic diagram of DA–MIP-coated gate FET. (**b**) Chemical composition of DA–MIP coated by SI-ATRP on Au electrode. (**c**) Equilibrium between PBA derivative and diol compound [74]. Copyright 2018 Elsevier.

**Figure 6:**
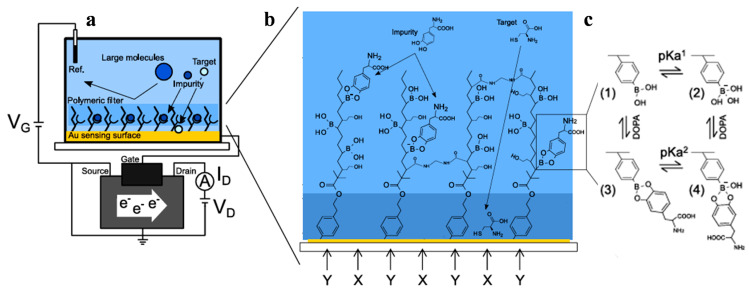
(**a**) Conceptual illustration of EG-Au-FET biosensor with nanofilter interface. (**b**) Conceptual design of nanofilter interface to trap L-DOPA and detect cysteine by Au electrode. (**c**) Binding equilibrium of L-DOPA and PBA [75]. Copyright 2019 American Chemical Society.

A field-effect transistor (FET) is a device that utilizes an electric field to control the conduction of charge carriers in a semiconductor channel between two electrodes. The gate electrode influences electrical conductivity by altering the electric field potential. It is a key electronic component used in many areas of the electronics industry. Kajisa et al. [74] reported a potentiometric sensor for dopamine (DA) detection with an extended Au-gate field-effect transistor (EG-Au-FET) (Figure 5). DA-templated MIP (DA–MIP) was grafted onto the Au electrode to form a biointerface for selective target recognition and then copolymerized with vinyl phenylboronic acid (vinyl-PBA) by SI-ATRP. The diol-binding between PBA and DA could induce a change in the surface potential, allowing for the detection of DA at a concentration of 0.04~20 μM. Meanwhile, Nishitani et al. [75] proposed a polymeric nanofilter biointerface for potentiometric determination of small molecules with the EG-Au-FET device (Figure 6). In this concept, a methacrylic acid (MAA)-based polymeric nanofilter was in situ-formed on the Au surface by cyclic voltammetry and photo-mediated SI-ATRP. Small molecules such as L-cysteine could reach the Au surface through the filter layer. In addition, PBA was copolymerized with the polymeric nanofilter to capture diol-containing species such as levodopa (L-DOPA) through boronic ester binding. The sensing electrode can detect different small molecules at the nanomolar level based on the change in the surface potential.

**Table 2:** Overview of ATRP-based materials for electrochemical detection of small molecules.

Method/Material	Target	Linear Range	Detection Limit	Ref.
MIP-IL@Au-CNS	Oxytetracycline	10^−2^–20 μM	5 nM	[62]
MWCNTs-*g*-HTPB-*b*-PABFC	Trichlorfon	1–10^6^ nM	35 nM	[63]
PNIPAM@SiO_2_	H_2_O_2_	0.1–333 mM	0.07 μM	[64]
MIP/AuNCs/Ni	Erythromycin	10–10^8^ pg/L	3.2 pg/L	[67]
Polymer/graphene	D-glucose	5 × 10^−5^–0.5 mM	1 nM	[68]
MGO-P(4-VBA)	Glucose	1–15 mM	39 μM	[73]
Vinyl-PBA-based MIP	Dopamine	0.04–20 μM	96 nM	[74]

Abbreviation: MIP-IL@Au-CNS, molecularly imprinted polymer–ionic liquids; MWCNTs, multi-walled carbon nanotubes; HTPB, hydroxyl-terminated poly butadiene; PNIPAM@SiO_2_, poly(N-isopropylacrylamide)-coated core–shell SiO_2_ microspheres; MIP, molecularly imprinted polymer; Au-CNS, gold nanoparticle-modified carbon nanospheres; MGO-P(4-VBA), magnetic Fe_3_O_4_ modified with graphene oxide poly(4-vinylphenylboronic acid); vinyl-PBA, vinyl phenylboronic acid.

#### 3.2.2. Optical Sensing

**Figure 7:**
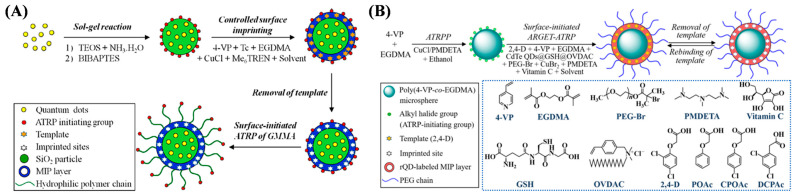
(**A**) Schematic protocol for the preparation of QD-labeled hydrophilic MIP microparticles [79]. Copyright 2016 American Chemical Society. (**B**) Schematic illustration for the preparation of red CdTe QD-labeled fluorescent 2,4-D-MIP microspheres with surface-grafted PEG brushes via one-pot SI-ARGET ATRP and chemical structures of some reagents utilized in this work [80]. Copyright 2024 Elsevier.

Optical sensors based on polymers and polymeric materials represent a revolutionary advancement in biomedical diagnosis and monitoring due to their unique flexibility, biocompatibility, and selective reactivity. ATRP has become one of the most widely used techniques for preparing multiblock copolymers and complex polymer structures. ATRP-based polymers and polymeric materials have been developed for the detection of small organic molecules with excellent performance. In addition, synthetic and natural macromolecules/polymers can be grafted onto the polymer skeleton to enhance the water solubility and biocompatibility of ATRP copolymers. Bismuth phosphate@GO and fluorescent polymers and MIPs labeled with dyes, quantum dots (QDs), and carbon dots have been prepared and used for sensing small molecules (Table 3) [76,77,78,79,80,81,82,83,84,85,86]. For example, Mardani et al. suggested that the fluorescent block copolymer poly(7-acryloyloxy 4-methylcoumarin-*r*-methyl methacrylate)-*b*-poly(dimethylaminoethyl methacrylate) formed by ATRP with a coumarin-containing ATRP initiator 7-(2-bromoisobutyryloxy)-4-methylcoumarin could be used for CO_2_ sensing [76]. The block copolymers could self-assemble into vesicular assemblies in an aqueous solution. In the presence of CO_2_, the thickness of the vesicular assemblies decreased, and their hydrodynamic radius increased, leading to an increase in the distance of coumarin moieties. The distance change of the coumarin moieties induced the change in the aggregation state of the copolymers and fluorescence intensity. In addition, many efforts have been put into creating organic fluorescent polymers and polymeric materials to address the key challenges in biological and medical applications. Yang et al. reported QD-labeled hydrophilic MIP microparticles for the detection of the antibiotic drug tetracycline (Tc) (Figure 7A) [79]. Alkyl bromide moieties were modified on the surface of CdTe QD–SiO_2_ composites for grafting Tc-imprinted polymers and poly(glyceryl monomethacrylate) brushes by SI-ATRP. Liu et al. [80] prepared hydrophilic QD-labeled fluorescent MIPs microspheres for fluorescent sensing of 2,4-dichlorophenoxyacetic acid, 2,4-D (Figure 7B). The microspheres were prepared through surface-initiated ARGET ATRP. Then, fluorescent MIPs were synthesized by one-step grafting CdTe QD-modified 2,4-D-MIPs with hydrophilic polyethylene glycol brushes onto the polymeric microspheres. In these two works, the fluorescence of QDs on the MIPs was selectively quenched by the target via the charge-transfer mechanism.

In addition to fluorescent assays, ATRP has also been used to prepare functional materials for colorimetric and SERS analysis of small molecules [87,88,89,90,91]. For example, Chen et al. reported Tc-imprinted Mn_3_O_4_ nanoparticles (DSMIP@Mn_3_O_4_) by the ATRP of ionic liquid monomers on the nanoparticle surface [87]. The DSMIP@Mn_3_O_4_ spheres with a core–shell structure exhibited high oxidase-like activity to catalyze the oxidation of 3,3′,5,5′-tetramethylbenzidine (TMB) into a blue product. Rebinding of Tc toward the DSMIP@Mn_3_O_4_ blocked the molecular channel and prevented the catalytic oxidation of TMB, thus allowing for the colorimetric assay of Tc in the linear range of 0.5–150 μM. Rong et al. prepared a SERS sensor by functionalizing GO with poly(2-(dimethylamino)ethyl methacrylate) (PDMAEMA) through the SI-ATRP technique [90]. The PDMAEMA-modified GO was quaternized to yield GO-*g*-qPDMAEMA and then integrated with AuNPs to form nanofilms through a water–oil interface assembly method. The nanofilms with remarkable SERS characteristics were used to detect antibiotics such as sulfamonomethoxine and enrofloxacin.

**Table 3:** Overview of ATRP-based materials for optical detection of small molecules.

Method/Material	Target	Linear Range	Detection Limit	Ref.
FMIP	2,4-D	0–25 µM	0.13 µM	[77]
FMIP	Fenvalerate	0–80 nM	0.068 nM	[78]
MIP-QD	Tetracycline	0.5–50 μM	0.14 μM	[79]
CdTe QD@MIP	2,4-D	1–10 μM	0.14 μM	[80]
Phenylene(vinylene) polymer	Picric acid	–	50 ppb	[81]
SiO_2_/ZnO/MIP	Cyhalothrin	1–80 μM	0.13 μM	[82]
Mn-doped ZnS QDs	Bifenthrin	5–50 μM	16.7 ng/mL	[83]
BiPO_4_@GO-MMIPs	Ciprofloxacin	39–740 μg/L	0.39 μg/L	[84]
SiO_2_-MPS@FMIP	λ-cyhalothrin	2–80 nM	3.7 nM	[85]
MAR@CD-MIP	2,4-D	18–72 μM	0.35 μM	[86]
DSMIP@Mn_3_O_4_	Tetracycline	0.5–150 μM	0.1 μM	[87]
Ag/CdTe/MIP	2,6-DCP	1–1000 nM	1 nM	[88]
Cu_2_O@Ag-MIP	Chlorophenol	10^−5^–1 mM	5.8 nM	[89]
GO-*g*-qPDMAEMA	Enrofloxacin	278–835 nM	1 nM	[90]

Abbreviation: FMIP, fluorescent molecularly imprinted polymer; 2,4-D, 2,4-dichlorophenoxyacetic acid; QD, quantum dot; BiPO_4_@GO, fluorescent bismuth phosphate@graphene oxide; MMIPs, magnetic nano-sized-molecularly imprinted polymers; MPS, 3-(methacryloxyl)propyl trimethoxysilane; DSMIP@Mn_3_O_4_, dual ionic liquid monomers on the surface of Mn_3_O_4_; 2,6-DCP, 2,6-dichlorophenol; GO-*g*-*q*PDMAEMA, graphene oxide functionalized with poly(2-(dimethylamino)ethyl methacrylate).

### 3.3. Bioimaging

**Figure 8:**
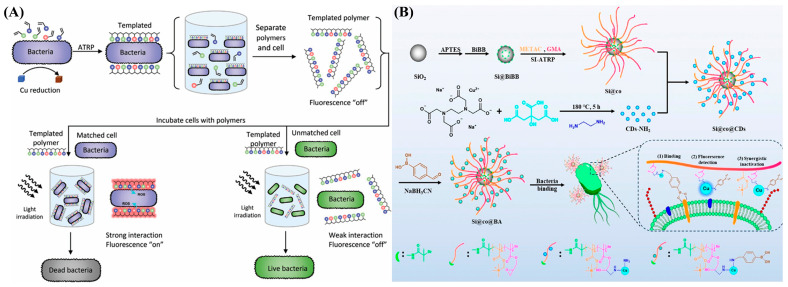
(**A**) Bacteria induce polymerization in monomer-catalyzed suspensions to generate a synthetic extracellular polymeric substance [97]. Copyright 2020 Wiley-VCH. (**B**) Schematic illustration for synthesis of multifunctional nanocomposite Si@co@BA and its application in binding, detection, and inactivation of bacteria [98]. Copyright 2025 American Chemical Society.

Optical imaging, especially fluorescence imaging, has attracted increasing interest in preclinical and clinical applications due to its advantages of non-invasiveness, real-time imaging, high resolution, no radiation-related risk, and low cost. Recent studies have demonstrated the feasibility of fluorescence imaging for in vivo imaging of tumor cells and bacterial and drug delivery using ATRP-based polymer materials [92,93,94,95]. However, the conventional fluorescence dyes usually show a high intrinsic background signal and readily suffer from the aggregation-caused quenching effect after polymerization. Aggregation-induced emission fluorogens (AIEgens) are nearly nonfluorescent in their molecular state but show strong luminescence in their aggregated state, showing great potential for clinical diagnostic and therapeutic applications [96]. Qi et al. prepared an acrylic polymer for self-selective binding and killing specific pathogenic bacteria with AIE characteristics using bacteria as the template by ATRP (Figure 8A) [97]. The monomers for bacterial templating included [2-(methacryloyloxy)ethyl]trimethyl ammonium chloride (TMAEMC), TMAEMC-TPAPy, and [2-(methacry loyloxy)ethyl]dimethyl-(3-sulfopropyl)ammonium hydroxide (DMAPS). TMAEMC is a permanent cation that can bind to the negatively charged bacterial cell surface. TPAPy is an AIE moiety that shows strong emission in its aggregated form. DMAPS is a zwitterionic sulfobetaine used to enhance polymer solubility and provide a spacer for cationic sections. The bacterium-templated polymers showed no fluorescence in an aqueous solution but exhibited strong emission after binding with the target bacteria. More interestingly, the AIEgens could serve as photosensitizers to produce reactive oxygen species (ROS) under light irradiation, endowing the polymers with excellent capability to selectively kill bacteria. Zhang et al. prepared multifunctional nanocomposites for bacterial binding, fluorescence imaging, and synergistic antibacterial treatment (Figure 8B) [98]. The nanocomposites were prepared by grafting cationic polymers with quaternary ammonium compounds onto SiO_2_ nanoparticles by ATRP, followed by incorporation of copper-doped CDs and modification of boronic acid. The cationic polymers and boronic acid groups were responsible for bacterial binding, and the CDs produced a high fluorescence signal even around bacteria, providing a novel approach for bacterial detection and synergistic treatment.

**Figure 9:**
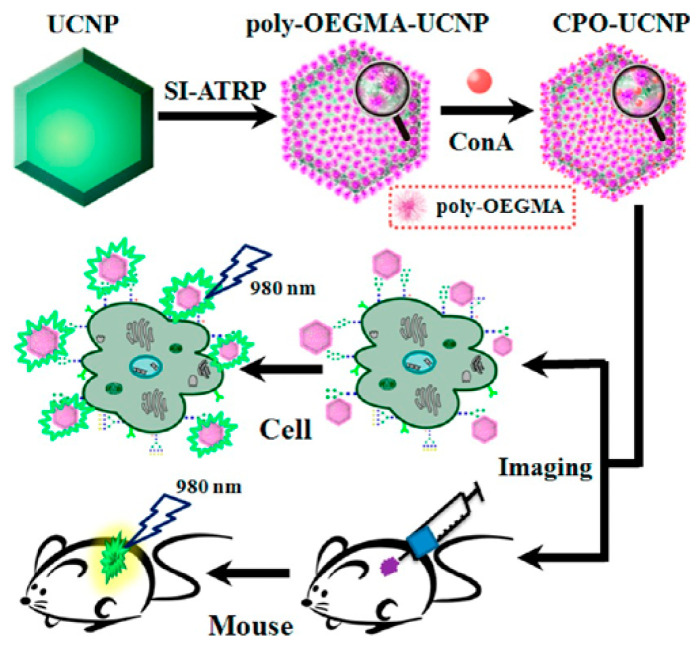
Schematic overview of the preparation procedure for poly-OEGMA-UCNPs, conjugation with ConA, and in vitro and in vivo imaging [100]. Copyright 2013 American Chemical Society.

Fluorescence imaging in the near-infrared (NIR) window can enable deep-tissue imaging with high resolution and improved contrast due to the reduced light scattering and tissue autofluorescence in this spectral region. He et al. prepared multifunctional magnetic nanoparticles for in vivo NIR imaging by grafting organic dyes with both excitation and emission in the NIR region on the surface of silica-coated iron oxides through the ATRP of PEGMA and GMA monomers [99]. In this method, biocompatible iron served as the catalyst for ARRP, and fluorophore CS_2_ was embedded into the polymer matrix by covalent coupling. Zhang et al. reported a method for labeling and imaging of cells using core–shell UCNPs prepared by the ATRP of hydrophilic oligo(ethylene glycol) methacrylate (OEGMA) monomers (Figure 9) [100]. The polymer layer could reduce the risk of crystal structure/surface morphology changes in the UCNPs and facilitate the immobilization of the lectin concanavalin A (ConA) to achieve glycan labeling. The resulting ConA-polyOEGMA-UCNPs (CPO-UCNPs) were successfully used to label highly metastatic hepatocellular carcinoma cells (HCCHM3) in vitro and image HCCHM3-inoculated mice in vivo.

**Figure 10:**
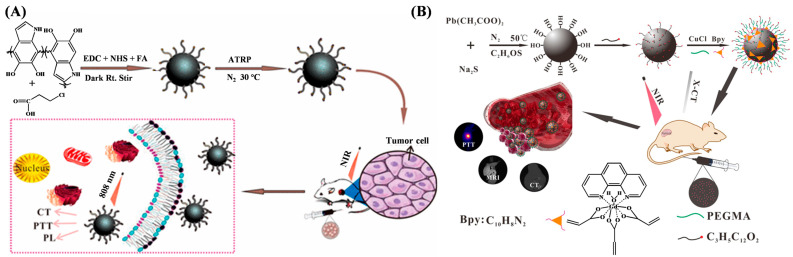
(**A**) Schematic illustration of FEDA nanoparticles for bioimaging-guided photothermal therapy [102]. Copyright 2019 American Chemical Society. (**B**) Schematic illustration of preparation processes of GCGLS nanoparticles and their use in dual-modality CT/MR imaging-guided photothermal ablation [105]. Copyright 2018 American Chemical Society.

Polydopamine (PDA) nanoparticles exhibit admirable photothermal properties and biocompatibility for biomedical applications. However, the NIR absorption and antibacterial performance of PDA remain insufficient. Zou et al. suggested that europium complexes could be grafted onto PDA nanoparticles by ATRP [101]. The nanoagents showed excellent X-ray computed tomography (CT) and photoluminescence (PL) properties, prominent NIR absorbance, and strong optical imaging efficiency. Zhang et al. reported PDA-based tumor-targeting multifunctional nanoparticles for CT/PL dual-mode bioimaging-guided photothermal therapy (PTT) (Figure 10A) [102]. The nanoparticles were prepared by modifying PDA with 3-chloropropionic acid (CPA) and folic acid (FA) via dehydration condensation, followed by grafting europium (III) complexes on the nanoparticle surface through ATRP. The resulting nanoagents, named FEDA, showed more outstanding imaging effects and a longer imaging time in contrast with the generally used reagents in clinical settings.

Imaging methods with multimodal contrast agents hold great potential for significant contributions in biomedical fields. Among modern imaging techniques, photoacoustic imaging (PAI) and magnetic resonance imaging (MRI) have gained much attention due to their non-invasive feature and mutually supportive characteristics. The ATRP technique has been used to prepare multimodal contrast nanoagents for imaging and synergistic therapy for tumors [103,104,105]. For example, Sun et al. prepared a zwitterionic polymer named PDS-PDI by ATRP as a PAI contrast agent and a photothermal agent [103]. Zou et al. fabricated a gadolinium(III) complex-grafted lead sulfide (GCGLS) theranostic nanoagent for CT and T1-weighted magnetic resonance (T1-MR) imaging-guided photothermal ablation (Figure 10B) [105]. The GCGLS nanoagents were prepared by grafting PEGMA and Gd(AA)_3_Phen on the 3-chloropropionic acid–lead sulfide (CPA-PbS) nanoparticle via ATRP. The nanoagent showed excellent CT and T1-MR imaging effects in vitro/vivo and could be used for tumor treatment in mice, with satisfactory results.

## 4. ATRP-Based Signal Amplification for Biosensors

### 4.1. Electrochemical Biosensors

#### 4.1.1. Electrochemical Sensing of Nucleic Acids

**Figure 11:**
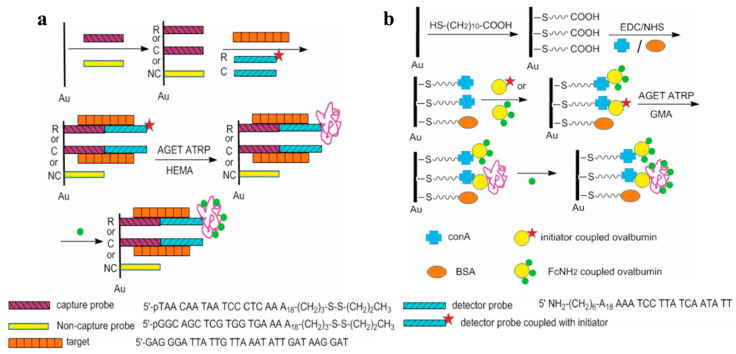
Polymerization-assisted electrochemical detection of DNA target through DNA hybridization (**a**) and protein binding (**b**) [106]. Copyright 2009 American Chemical Society.

To achieve ultrasensitive detection, a variety of materials or strategies, including nanomaterials, enzymatic catalysis, and nucleic acid amplification techniques, have been integrated into electrochemical biosensors for signal amplification. For example, rolling circle amplification (RCA), a powerful isothermal nucleic acid amplification technique, can produce thousands of repeating DNA sequences in the presence of circular templates with the aid of DNA polymerase. When antibodies are conjugated with DNA strands and form circular templates in the presence of antigens, the types of targets can expand from nucleic acids to proteins, exosomes, and cells. However, the electroactivity of nucleic acids is low, and extra electroactive species are usually required to provide an enhanced electrochemical signal. Due to its unique characteristics, ATRP can provide site-specific grafting to various biomacromolecules, including peptides, DNA, PNA, and antibodies. Furthermore, monomers can be modified with electrochemical reporters, especially ferrocene (Fc). A large number of electroactive units in the in situ-formed polymers can greatly amplify the electrochemical response. Therefore, ATRP techniques have been widely integrated with electrochemical biosensors for signal-amplified detection of nucleic acids, proteins, enzymes, and antigens. In 2009, He’s group first suggested that ATRP could be used for the design of biosensors by providing multiple binding sites to immobilize signal tags (Figure 11) [106,107]. The capture of ATRP initiator-labeled DNA or protein allows for the formation of an extended polymer as the carrier to couple multiple signal tags, such as electroactive aminoferrocene (FcNH_2_). Initiation of the polymerization reaction occurs via the chemical reduction of Cu(II) to Cu(I). It is crucial to emphasize that this reduction process does not directly generate free radicals; instead, its function is to regenerate the Cu(I)/L activator complex (e.g., the Me_6_TREN-Cu(I) complex). As the true active catalytic species, the regenerated Cu(I)/L complex reacts with alkyl halide to produce a free radical source that can initiate the polymerization reaction. Recently, this concept was used to develop various sensing platforms, such as DNA biosensors, aptasensors, and immunosensors, by grafting electroactive or dye monomers on the sensor interface by different ATRP methods (Table 4).

**Figure 12:**
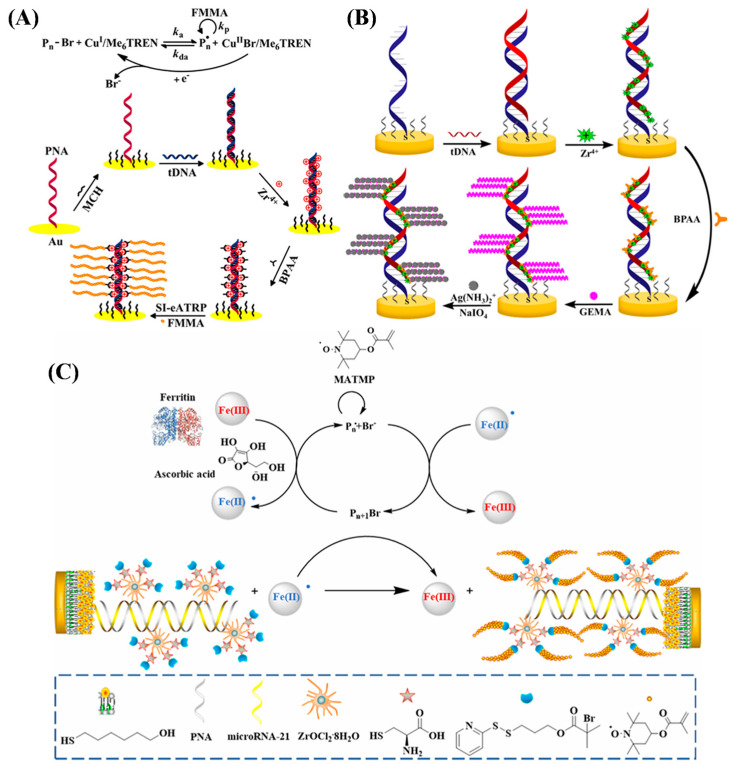
(**A**) Principle of the dnGOP-based electrochemical detection of DNA [108]. Copyright 2017 American Chemical Society. (**B**) Schematic illustration of the DNA biosensor based on SI-eATRP signal amplification and DNA-templated silver particles [112]. Copyright 2019 American Chemical Society. (**C**) Schematic illustration of lung cancer-related microRNA-21 detection that can be tracked via sensor analysis mediated by Ft-ATRP [116]. Copyright 2023 American Chemical Society.

Among the family of ATRP reactions, eATRP stands out due to its ability to precisely control the initiation, cessation, and reinitiation of the polymerization process, controllable reaction rate, and high tolerance to O_2_. Under a negative potential, Cu^2+^ can be reduced to Cu^+^ to trigger SI-ATRP for the in situ formation of electroactive polymers with different initiators and monomers [108,109,110,111]. Typically, Hu et al. developed an electrochemical DNA biosensor based on electrochemically mediated SI-ATRP (SI-eATRP) for the de novo growth of polymers (dnGOP) (Figure 12A) [108]. Target DNA (tDNA) captured by peptide nucleic acid (PNA) provided numerous phosphate groups for the attachment of ATRP initiators on the electrode surface through the phosphate–Zr^4+^–carboxylate interactions. Cu^I^/tris(2-dimethylaminoethyl)amine (Cu^I^/Me_6_TREN) was in situ electrochemically generated as the activator to trigger the SI-eATRP of FMMA monomers. The formed dnGOPs produced an amplified electrochemical signal for tDNA detection in the concentration range from 0.1 fM to 0.1 nM. In addition to the polymerization of electroactive small molecules, the resulting polymers could provide numerous aldehyde groups for the deposition of silver nanoparticles (AgNPs) through the silver mirror reaction, producing a well-defined electrochemical signal from the oxidation of Ag^0^ into Ag^+^ [112,113]. For example, Sun et al. proposed an ATRP-based signal amplification method for DNA detection with DNA-templated AgNPs as the electrochemical signal reporters (Figure 12B) [112]. Glycosyloxyethyl methacrylate (GEMA) was linked to the PNA/DNA duplex by ATRP. Then, the polysaccharide in GEMA was oxidized into an aldehyde group by NaIO_4_, which allowed for the in situ formation and deposition of AgNPs through the silver mirror reaction.

In addition to the electrochemical technique, ATRP can be mediated by other methods, such as chemical reduction and photocatalysis. The air-stable deactivators, metal ions (e.g., Cu^2+^) and metalloproteins (e.g., hemoglobin and ferritin), can be chemically reduced into activators to trigger the ATRP reaction [114,115]. Ma et al. reported an electrochemical biosensor for miRNA-21 detection by ferritin-enhanced ATRP (Ft-ATRP) (Figure 12C) [116]. The phosphate groups in miRNA-21 captured by the PNA probe were coordinated with Zr^4+^ to immobilize cysteine for the attachment of multiple ATRP initiators. Then, ferritin-mediated aggregation of hydrophilic methacryloyloxy-2,2,6,6-tetramethylpiperidine 1-oxyl free radical (MATMP) monomers with a well-defined electrochemical signal on the electrode surface was realized.

**Figure 13:**
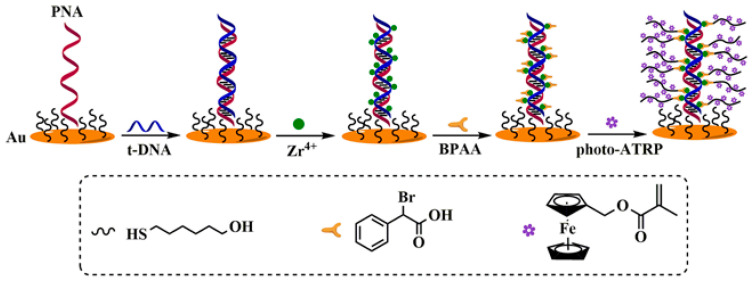
Proposed mechanism of DNA sensor based on photo-ATRP [117]. Copyright 2023 Elsevier.

Photo-ATRP can be activated by photoinitiators such as I2959, rose bengal (RB), 10-phenylphenothiazine (PTH), rhodamine 6G (R6G), and CuFe_2_O_4_ to produce free radicals for grafting numerous electroactive probes onto the electrode surface [117,118,119,120,121,122]. Yu et al. reported an electrochemical DNA biosensor using the photocatalyst PTH to activate the initiator α-bromophenylacetic acid (BPAA) under 365 nm UV light irradiation, generating active radicals [117]. Hu et al. [118] reported an RB-mediated photo-ATRP method for sensitive detection of DNA under the excitation of blue light with β-nicotinamide adenine dinucleotide (NADH) as the electron donor. In these works, FMMA molecules were employed as the monomers to achieve the photo-ATRP and form electroactive polymer chains on the electrode surface for signal amplification (Figure 13). In order to further improve the sensitivity, GO and AuNPs could be connected onto the DNA strand by phosphate–Zr^4+^–carboxylate chemistry or Au-S interactions, introducing plenty of ATRP initiators for signal amplification [123,124]. However, the aforementioned studies were usually conducted under oxygen-free conditions because oxygen is a critical concern for ATRP implementation. This is primarily attributed to the fact that oxygen can readily react with active radicals in the polymerization system, forming stable peroxyl radicals that further terminate the polymerization process or induce uncontrolled growth of polymer chains. Notably, the groups of Matyjaszewski and others have conducted valuable studies on oxygen-tolerant ATRP techniques [125,126]. These works provide a feasible way to break the dependence of traditional ATRP-based sensors on oxygen-free environments, offering key technical references for the practical application of ATRP techniques in biosensors. In addition, the flashlight of smartphones can trigger the photo-RDRP and ATRP based on a dual catalytic system with the CuBr_2_/tris(2-pyridylmethyl)amine complex and different fluorescent dyes (e.g., eosin Y, fluorescein, and riboflavin) as the catalysts [127], which is an interesting future endeavor for analyte detection.

**Figure 14:**
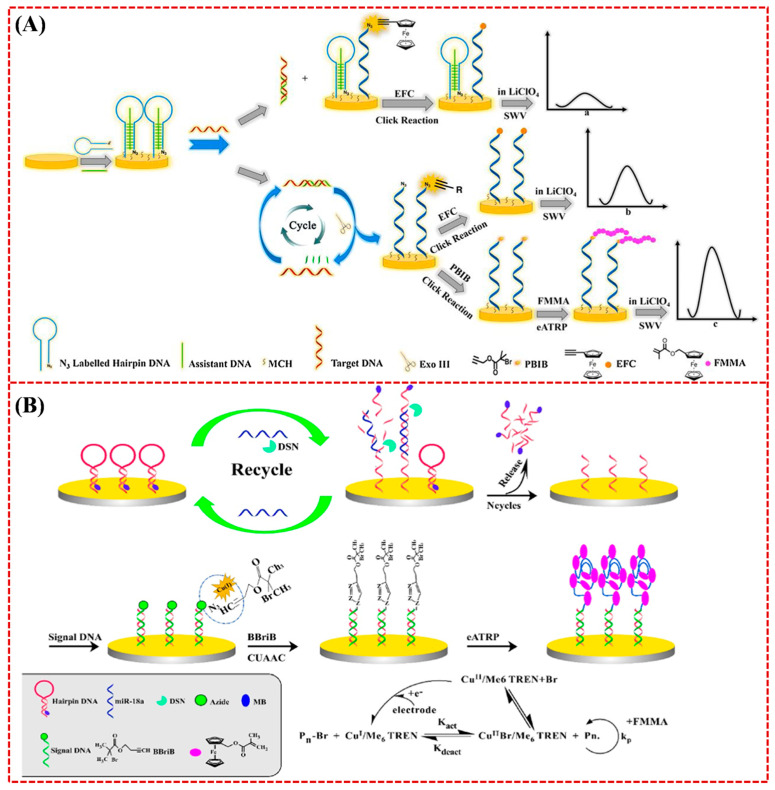
(**A**) The principle of nucleic acid detection based on Exo III-mediated target cycling and eATRP double signal amplification [129]. Copyright 2021 Elsevier. (**B**) Schematic of the fabrication route to the biosensor [132]. Copyright 2021 Elsevier.

In addition to phosphate–Zr^4+^–carboxylate chemistry, click chemistry has also been commonly used to link the ATRP initiator on the electrode surface by using an azide (N_3_)-labeled probe [128]. In this method, ATRP can be readily integrated with other techniques to achieve multi-signal amplification, such as exonuclease III (Exo III) and duplex-specific nuclease (DSN)-assisted target cycling and enzyme-free isothermal amplification strategies, including catalytic hairpin assembly (CHA), strand displacement amplification (SDA), and hybridization chain reaction (HCR) [129,130,131]. For example, Sun et al. reported an electrochemical biosensor based on the dual-signal amplification of Exo III-mediated target cycle and eATRP (Figure 14A) [129]. In that work, triple-helical DNA labeled with a N_3_ tag was immobilized on a gold electrode to capture target DNA. Exo III-mediated target cycle was triggered to expose the N_3_ tag on the electrode surface, allowing for the conjugation of initiator propargyl-2-bromoisobutyrate (PBIB) for the eATRP of FMMA monomers. Rezaei et al. reported a ratiometric electrochemical biosensor for the determination of microRNA-18a (miR-18a) based on the signal amplification of DSN-assisted target recycling and eATRP (Figure 14B) [132]. The formation of miR-18a/DNA duplexes induced the cleavage of MB-labeled DNA capture probes through DSN-assisted target recycling, leading to the release of MB from the electrode surface (signal-off). The remaining piece of DNA capture probes could hybridize with N_3_-DNA signal probes to allow for the conjugation of 3-butynyl-2-bromoisobutyrate (BBriB) through the click reaction with N_3_, initiating the eATRP of FMMA on the electrode surface (signal-on). The on–off current ratio (*I*_FMMA_/*I*_MB_) was proportionate to the miR-18a concentration in the range from 100 aM to 50 pM.

**Figure 15:**
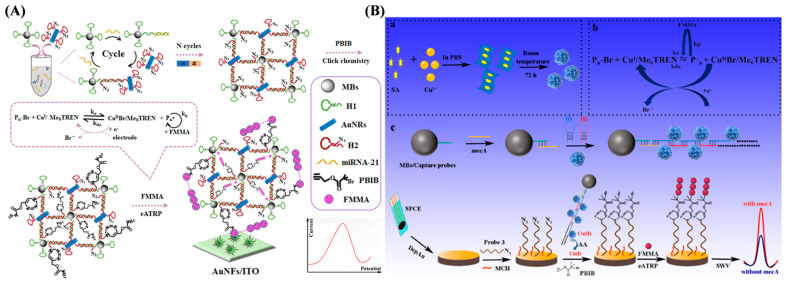
(**A**) Schematic diagram of multi-amplified platform for miRNA-21 detection [133]. Copyright 2020 American Chemical Society. (**B**) Schematic diagram of synthesis process of SA@Cu HNFs (**a**) and principle of eATRP (**b**), as well as magnetic separation-based electrochemical biosensing platform (**c**) [134]. Copyright 2025 Elsevier.

In addition, Peng et al. reported an enzyme-free electrochemical biosensor for miRNA-21 detection by combining a magnetic separation system with strand displacement amplification (SDA) and eATRP (Figure 15A) [133]. After the target-triggered SDA, a number of N_3_-DNA probes were anchored on the surface of magnetic nanobeads (MBs). The magnetic networks were then captured by AuNFs/ITO to achieve the conjugation of PBIB for initiating the eATRP of electroactive FMMA monomers. The excellent anti-interference ability and high sensitivity and specificity of this method were attributed to the use of a magnetic separation system and a AuNF-modified sensing electrode, as well as SDA- and eATRP-based multi-signal amplification. Recently, Huo et al. reported an electrochemical method for mecA gene detection by integrating a magnetic separation system with the signal amplification of HCR and eATRP (Figure 15B) [134]. To weaken the interference of complex matrices, the mecA gene was captured and enriched by a magnetic system, triggering the formation of long biotin-containing DNA polymers by the signal amplification of HCR. Then, streptavidin–copper hybrid nanoflowers (SA@Cu HNFs) were attached to the polymers through the avidin–biotin interactions. Cu(I) released from SA@Cu HNFs served as a catalyst and signal transduction modulator to promote the click reaction between PBIB and N_3_ on the electrode surface, initiating the in situ eATRP of electroactive FMMA monomers and achieving the signal-amplified detection of the *mecA* gene.

#### 4.1.2. Electrochemical Aptasensors

**Figure 16:**
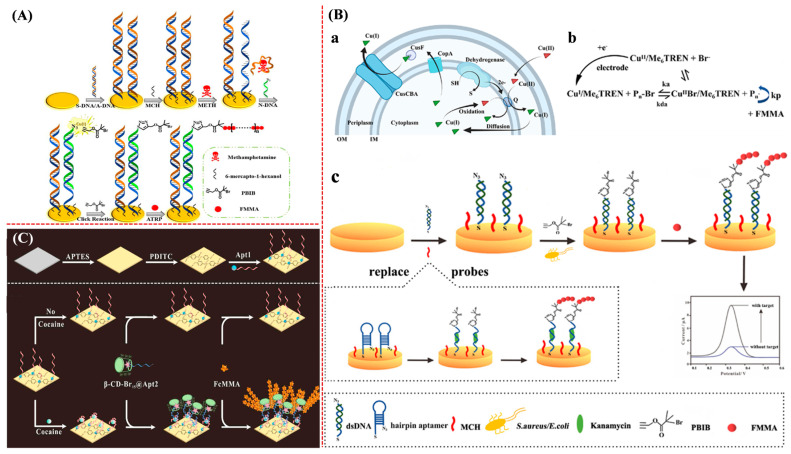
(**A**) Principle of METH sensor preparation process [138]. Copyright 2022 Elsevier. (**B**) Schematic representation of (**a**) copper reduction system in bacteria, (**b**) principle of eATRP (P_n_-Br rate constant, k_a_ = alkyl bromide, k_da_ = activation rate constant, and k_p_ = propagation rate constant), and (**c**) electrochemical biosensor for bacterial analysis and antibiotic detection [139]. Copyright 2022 Elsevier. (**C**) Illustration of β-CD-Br-15-based electrochemical biosensor for cocaine detection based on host–guest chemistry [140]. Copyright 2021 Elsevier.

Electrochemical aptasensors can be designed by using double-stranded or hairpin DNA probes to recognize the targets. The interaction between the aptamer and the target can facilitate the conjugation of the initiator as the linker to trigger the ATRP process for signal amplification [21,135,136,137]. Sun et al. reported a competitive electrochemical aptasensor for methamphetamine (METH) detection based on an ATRP signal amplification strategy (Figure 16A) [138]. METH could bind with the aptamer from the double-stranded DNA, releasing the complementary DNA strand to hybridize with N_3_-modified DNA on the electrode surface. Based on the click chemistry and ATRP, the electroactive FMMA monomers were polymerized into long-chain polymers to produce an amplified electrochemical signal. In addition, bacteria can initiate click chemistry by reducing Cu^II^ to Cu^I^. Li et al. designed an electrochemical biosensor for monitoring the levels of *Staphylococcus aureus* (*S. aureus*) and *Escherichia coli* (*E. coli*) by integrating click chemistry with ATRP for signal amplification (Figure 16B) [139]. In order to improve the selectivity and anti-interference ability, the target bacteria were pre-extracted and concentrated using aptamer-modified magnetic beads. The method can be used for rapid drug resistance analysis by incubation of bacteria with an anti-bacterial drug in advance. In addition, aptasensors have been designed for antibiotic residue detection by substituting the target-specific recognition element. The hairpin N_3_-DNA probe immobilized on the electrode surface could be opened by binding with the antibiotic kanamycin, exposing the N_3_ group to react with the initiator PBIB for the ATRP of FMMA monomers. Wang et al. proposed a sandwich electrochemical aptasensor based on the host–guest chemistry and ATRP signal amplification (Figure 16C) [140]. The cocaine aptamer (Apt1) was fixed on the indium–tin–oxide electrode for specific target capture. Then, Fc-DNA (Apt2) was attached to the electrode surface by binding with cocaine. The ATRP initiator (β-CD-Br-15) was then anchored on Apt2 by the β-CD-Fc host–guest interaction, further triggering the ATRP of FMMA monomers. Additionally, in order to further improve the detection sensitivity, ATRP can be integrated with other materials or methods such as nanomaterials, enzymatic catalysis, and CHA to achieve dual-signal amplification for aptasensors [141].

**Figure 17:**
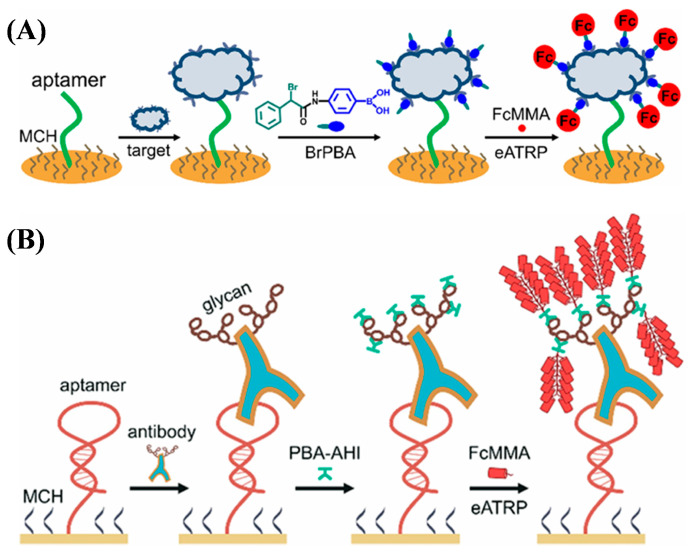
(**A**) Principle of eATRP-based electrochemical aptasensing of tumor biomarkers [146]. Copyright 2022 American Chemical Society. (**B**) Principle of glyGPC-based electrochemical detection of antibody pharmaceuticals [147]. Copyright 2024 American Chemical Society.

Aptasensors can be designed in a sandwich format by using an additional aptamer or synthetic material as the recognition element. By labeling the recognition element with an initiator, an electroactive polymer can be formed on the electrode surface by different ATRP techniques [142]. For example, boronic acid-containing initiators have been used as the linkers to recognize captured glycoproteins and initiate the eATRP or photo-ATRP of electroactive monomers for signal amplification [143,144,145]. Typically, Hu et al. developed an electrochemical aptasensor for the detection of glycoproteins based on boronic acid recognition and ATRP signal amplification (Figure 17A) [146]. The ATRP initiator (4-(2-bromo-2-phenylacetylamino)phenyl)boronic acid (BrPBA) captured by the glycoproteins on the aptamer-modified electrode triggered the ATRP reaction. In addition, based on boronic acid-based recognition and ATRP-based signal amplification, the antibody drug trastuzumab (Herceptin) was determined with a detection limit of 71.5 pg/mL (Figure 17B) [147]. The glycan-initiated site-directed signal amplification strategy exhibited great promise in the detection of diol-containing species.

**Figure 18:**
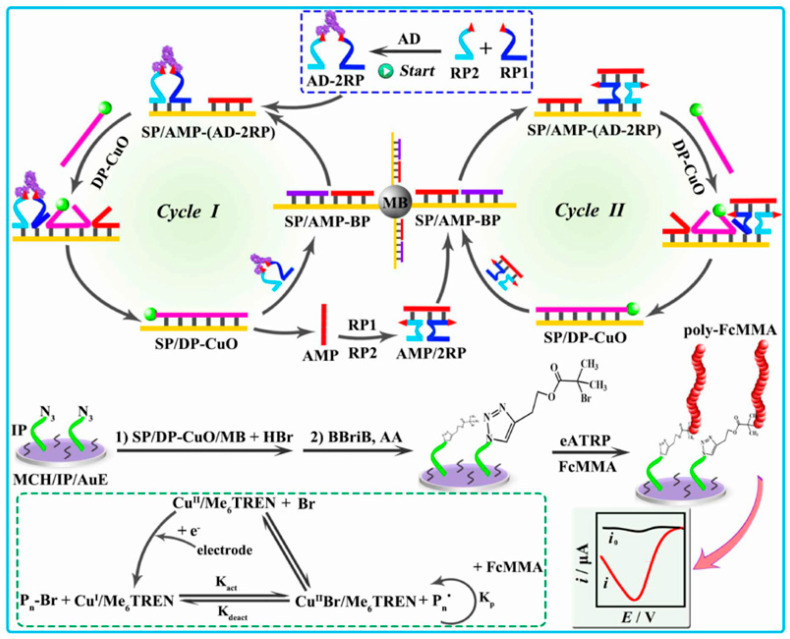
Schematic description of the programmable DNA nanomachine based on the combination of antibody-responsive eATRP amplification with cascaded strand displacement reaction [148]. Copyright 2024 American Chemical Society.

DNA nanomachines are nanorobots made entirely or partially of DNA. They can switch between defined molecular conformations and serve as sensing, computing, actuating, or therapeutic nanodevices. Dou et al. designed an electrochemical biosensor for the detection of antibodies by integrating a programmable DNA nanomachine with eATRP for signal amplification [148]. As shown in Figure 18, two DNA probes (RP1 and RP2) were conjugated with anti-Dig to form the AD-2RP complex for binding to the biotinylated substrate probe (SP) and antibody-mimic probe (AMP). This initiated the strand displacement reaction in the presence of displacement probe (DP)-modified CuO nanoparticles (DP-CuO) and induced the formation of SP/DP-CuO and the release of AMP and AD-2RP for recycling use (cycle I). The AMP-2RP complex formed between the hybridization of AMP with RP1 and RP2 could hybridize with SP through the terminal toehold, displacing the block probe (BP) strand to expose the toehold in the middle region. Meanwhile, the AMP-2RP complex could function with the SP/AMP-BP to perform a similar toehold-mediated strand displacement reaction (cycle II). After the cascaded recycling, a large number of SP/DP-CuO complexes were attached onto the surface of streptavidin-modified magnetic beads (MBs) through avidin–biotin interactions. Cu^ΙI^ was then released and reduced to Cu^I^ to catalyze the azide–alkyne cycloaddition reaction for the ATRP of electroactive FMMA monomers on the electrode surface.

#### 4.1.3. Electrochemical Sensing of Enzymes

**Figure 19:**
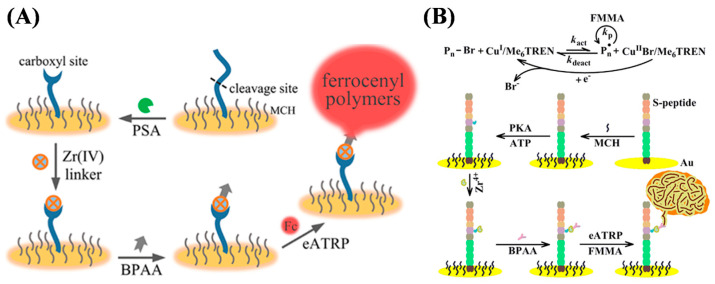
(**A**) Schematic illustration of cleavage-based electrochemical PSA biosensor [156]. Copyright 2020 American Chemical Society. (**B**) Schematic illustration of polymerization-based electrochemical detection of PKA activity [153]. Copyright 2018 Elsevier.

Enzyme assays are important in many applications, including clinical diagnosis, functional proteomics, and drug discovery. Research efforts have been made on the development of enzyme biosensors to detect their activities and levels and screen potential inhibitors. Recently, proteases, tyrosinase, and protein kinases have been determined based on the signal amplification of ARGET ATRP, photo-ATRP, and eATRP for the in situ formation of electroactive FMMA polymers [149,150,151,152,153,154,155,156]. In these works, the initiators were usually linked to the activated sites through the carboxylate–Zr^4+^–carboxylate or carboxylate–Zr^4+^–phosphate interactions. For example, Hu et al. developed an electrochemical biosensor for the detection of the protease prostate-specific antigen (PSA) based on the target-induced cleavage of the peptide substrate and eATRP signal amplification (Figure 19A) [156]. Enzymatic cleavage of the carboxyl-free peptide substrate on the electrode surface led to the generation of a carboxyl group that can react with the alkyl halide initiator BPAA through the carboxylate–Zr^4+^–carboxylate interaction. Then, the eATRP of FcMMA monomers on the electrode surface resulted in the formation of high-density ferrocenyl polymers for the signal-amplified output. Based on the carboxylate–Zr^4+^–phosphate chemistry, Hu et al. also reported the electrochemical detection of protein kinase A (PKA) by eATRP signal amplification (Figure 19B) [153]. In addition to eATRP, photo-ATRP has also been used to design electrochemical biosensors for the detection of enzyme activity with Zr^4+^ as the linker. Typically, Yu et al. developed an electrochemical thrombin biosensor using perylene as the photocatalyst to mediate the polymerization of FMMA for signal amplification [157].

#### 4.1.4. Electrochemical Immunoassays of Proteins and Others

**Figure 20:**
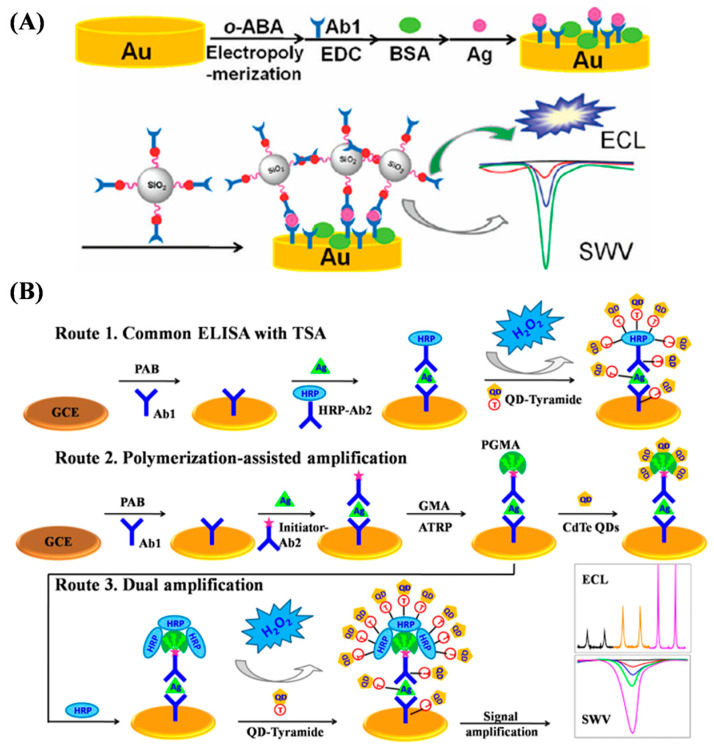
(**A**) Schematic representation of sandwich immunoassay with Si/PGMA/QD as label [162]. Copyright 2011 American Chemical Society. (**B**) Schematic representation of typical tyramide signal amplification based on common ELISA using QD–tyramide conjugate as label (Route 1the polymerization-assisted amplification in sandwich immunoassay via SI-ATRP and subsequent direct binding of CdTe QDs (Route 2), and sandwich immunoassay using QD–tyramide conjugate as label via SI-ATRP and tyramide signal amplification (Route 3) [171]. Copyright 2012 American Chemical Society.

Electrochemical immunoassays use antibodies as capture and/or recognition elements to quantitatively determine the electrical signals generated by the immunoreaction events between antibodies and antigens. Such methods can be categorized based on the type of output signals, such as amperometric, potentiometric, and conductometric immunoassays. Polymeric hybrid materials such as palladium nanocages, MWCNT, GO, and silica nanosphere have been prepared by different ATRP methods and then modified with target-specific recognition antibodies as the signal reporters for electrochemical and ECL immunoassays [158,159,160,161]. For example, Yuan et al. developed a sandwich ECL immunoassay platform for the detection of tumor necrosis factor-alpha (TNF-R) with a QD–polymer-functionalized silica nanosphere as the signal label (Figure 20A) [162]. The silica nanosphere was coated with the glycidyl methacrylate ATRP polymer (PGMA) as the carrier to bind CdTe QDs via the ring-open reaction. In addition, polymers can be in situ-formed on the electrode surface by ATRP to amplify the signals of immunoassays with initiator-labeled antibodies as the recognition elements [163,164,165,166,167,168,169,170]. The yielded polymers could serve as electroactive probes or HRP carriers for the direct or enzymatic signal readout. Typically, Yuan et al. reported an ECL immunosensor based on the dual-signal amplification of tyramide and polymerization (Figure 20B) [171]. The PGMA polymer was in situ-formed on the electrode surface captured with the initiator-labeled antibody. The formation of long-chain PGMA polymer provided numerous epoxy groups for the coupling of HRP molecules and the loading of QD–tyramide conjugates. The ECL and voltammetric signal increased by 9.4- and 10.5-fold in contrast with that without the dual-signal amplification, respectively.

**Table 4:** Overview of ATRP-based signal amplification for electrochemical biosensors.

Biosensor	Analyte	Signal Amplification	Linear Range	Detection Limit	Ref.
Nucleic acid sensing	DNA	AGET ATRP	0.1–1000 nM	15 pM	[106]
	DNA	AGET ATRP	1–100 nM	1 nM	[107]
	DNA	eATRP	10^−4^–0.1 nM	0.072 fM	[108]
	DNA	eATRP	10^−5^–10 pM	0.2 aM	[109]
	DNA	eATRP	10^−7^–0.1 nM	9.04 aM	[110]
	DNA	eATRP	10^−4^–10 pM	25 aM	[111]
	DNA	eATRP	10^−5^–10 pM	4.725 a M	[112]
	DNA	eATRP	10^−6^–10 pM	0.487 aM	[113]
	DNA	eATRP	10^−6^–1 nM	0.47 fM	[114]
	DNA	Hb-ATRP	10^−2^–10 nM	15.96 fM	[115]
	miRNA-21	Ft-ATRP	10^−2^–100 pM	6.03 fM	[116]
	DNA	Photo-ATRP	10^−5^–10 pM	79 aM	[117]
	DNA	Photo-ATRP	1–10^5^ fM	0.115 fM	[118]
	DNA	Photo-ATRP	10^−5^–1 nM	3.16 fM	[119]
	DNA	Photo-ATRP	10^−4^–10 pM	1.98 aM	[120]
	TMV RNA	Photo-ATRP	0.01–10 nM	3.5 fM	[121]
	RNA	PET-ATRP	10^−6^–0.1 nM	0.12 fM	[122]
	DNA	eATRP	10^−6^–0.1 fM	0.213 aM	[123]
	miRNA-141	ATRP	10^−5^–10 pM	3.23 aM	[124]
	TMV RNA	eATRP	10^−4^–10 nM	2.61 fM	[128]
	DNA	eATRP	10^−2^–10 fM	1.954 aM	[129]
	miRNA-21	Cu-ATRP	10^−8^–0.1 nM	4.96 aM	[131]
	miR-18a	eATRP	10^−4^–50 pM	2.5 aM	[132]
	miRNA-21	eATRP	10^−9^–1 nM	0.32 aM	[133]
	mecA gene	eATRP	10^−4^–10 pM	0.06 fM	[134]
Aptasensor	ERα	AGET ATRP	10^−5^–10 ng/mL	2.56 fg/mL	[135]
	Bisphenol A	eATRP	10^−5^–100 nM	59 aM	[136]
	Acetamiprid	ATRP	7 × 10^−2^–300 ng/mL	19.26 pg/mL	[137]
	METH	eATRP	10^−3^–100 nM	17 fM	[138]
	*S. aureus* and *E. coli*	eATRP	10^2^–10^7^ CFU/mL	4 and 6 CFU/mL	[139]
	Cocaine	AGET ATRP	10^−5^–10 mg/mL	0.0335 ng/mL	[140]
	Digoxin	eATRP	1–40 pM	0.59 pM	[141]
	CEA	eATRP	10^−3^–10^2^ ng/mL	70.17 fg/mL	[142]
	HER2	AGET ATRP	10^−5^–10 µg/mL	0.39 pg/mL	[143]
	LPS	Photo-ATRP	10^−3^–0.1 pg/mL	0.25 fg/mL	[144]
	AFP	eATRP	10^−3^–1 ng/mL	0.32 pg/mL	[146]
	Trastuzumab	eATRP	5 × 10^−2^–50 ng/mL	71.5 pg/mL	[147]
	Anti-Dig	eATRP	10^−3^–200 nM	1.5 pM	[148]
Enzyme sensing	Tyrosinase	Photo-ATRP	0.06–1 U/L	0.048 U/L	[149]
	ALP	AGET ATRP	20–200 mU/mL	1.64 mU/mL	[150]
	ALP	AGET ATRP	5–100 mU/mL	1.71 mU/m	[151]
	ALP	Photo-ATRP	10–150 mU/mL	2.12 mU/mL	[152]
	Protein kinase	eATRP	0–140 mU/mL	1.63 mU/mL	[153]
	MMP-2	eATRP	10^−3^–80 pM	0.53 fM	[154]
	Trypsin	eATRP	30–210 μU/mL	16 μU/mL	[155]
	PSA	eATRP	10^−5^–10 nM	3.2 fM	[156]
	Thrombin	Photo-ATRP	10^−5^–1 ng/mL	4 fg/mL	[157]
Immunosensor	CA153	ATRP	10^−2^–120 U/mL	0.003 U/mL	[158]
	CEA, AFP	PET-ATRP	1.63 × 10^−4^–163, 10^−4^–100 ng/mL	56.1 fg/mL and 32.8 fg/mL	[159]
	AFP	AGET ATRP	10^−4^–100 ng/mL	0.08 pg/mL	[160]
	AFP	SI-ATRP	25–50,000 pg/mL	0.183 pg/mL	[161]
	TNF-α	ATRP	10^−4^–1 μg/mL	3 pg/mL	[162]
	DR1	ATRP	5 × 10^−4^–5 × 10^2^	0.159 pg/mL	[163]
	DR1	ATRP	10^−4^–10^2^ ng/mL	2.91 fg/mL	[164]
	CEA, AFP, CA125, and CA153	AGET ATRP/HRP	0.01–100, 0.01–100, 0.05–100, 5 × 10^−2^–100 ng/mL	0.01, 0.01, 0.05, 0.05 ng/mL	[165]
	PSA	AGET ATRP/HRP	5 × 10^−3^–20 ng/mL	1.3 pg/mL	[166]
	IgG	SI-ATRP	5–70 ng/mL	0.3 ng/mL	[167]
	CYFRA 21–1	Photo-ATRP	10^−5^–1 ng/mL	5.8 fg/mL	[168]
	CYFRA 21–1	eATRP	10^−9^ fg/mL–1 μg/mL	0.8 fg/mL	[169]
	CA19-9	ATRP	10^−4^–100 U/mL	39 µU/mL	[170]
	IgG	ATRP/HRP	10^−3^–10 ng/mL	0.73 and 0.09 pg/mL	[171]

Abbreviation: TMV, tobacco mosaic virus; ERα, estrogen receptor α; METH, methamphetamine; *S*. *aureus*, Staphylococcus aureus; *E*. *coli*, Escherichia coli; anti-Dig, anti-digoxin antibody; CEA, carcinoembryonic antigen; LPS, lipopolysaccharide; AFP, alpha-fetoprotein; TNF-α, tumor necrosis factor-alpha; ALP, alkaline phosphatase; MMP-2, matrix metalloproteinase 2; PSA, prostate-specific antigen; CA153, carbohydrate antigen 153; DR1, down-regulator of transcription 1; HER2, human epidermal growth factor receptor 2; CA125, cancer antigen 125; Cyfra21-1, cytokeratin fragment 21-1; CA19-9, carbohydrate antigen 19-9.

### 4.2. Optical Biosensors

**Figure 21:**
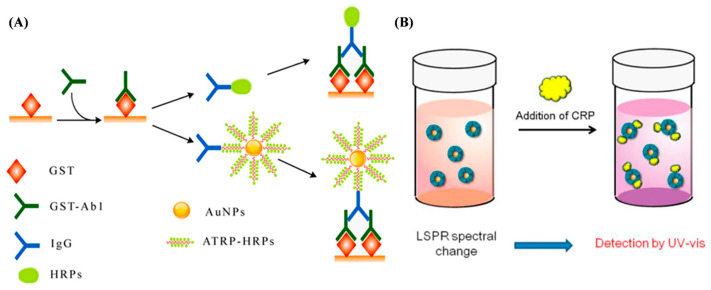
(**A**) Schematic diagram of conventional and signal-amplified ELISA [175]. Copyright 2014 American Chemical Society. (**B**) CRP nanosensing based on LSPR property of PMPC-*g*-AuNPs by UV–vis [176]. Copyright 2014 American Chemical Society.

Optical biosensors show immense potential and offer extraordinary possibilities for biosensing due to their high sensitivity, reusability, and ultrafast sensing capabilities. They mainly include colorimetry, fluorescence, SPR, SERS, etc. ATRP techniques have been used to synthesize different polymers and polymeric materials for colorimetry, SPR, and fluorescence biosensors [172,173,174]. The resulting products can be used to modify the sensing interface for molecular immobilization and serve as the labels for signal amplification. For example, Chen et al. suggested that ATRP polymer-modified AuNPs can be used as the carriers to load an abundance of HRP labels for ELISA (Figure 21A) [175]. The detection limit of the proposed ELISA was lower than that of conventional ELISA by a factor of 81. Kitayama et al. reported a colorimetric biosensor based on the LSPR of AuNPs by using the ATRP polymer as an artificial protein recognition layer (Figure 21B). The poly(2-methacryloyloxyethyl phosphorylcholine) polymer was grafted on AuNPs (PMPC-*g*-AuNPs) to bind the target protein with CRP as an example. The target concentration change was determined based on the shift in the LSPR spectra derived from the permittivity change of polymerized AuNPs. The work provided a foundation for the design of various biosensors to determine other protein biomarkers [176].

**Figure 22:**
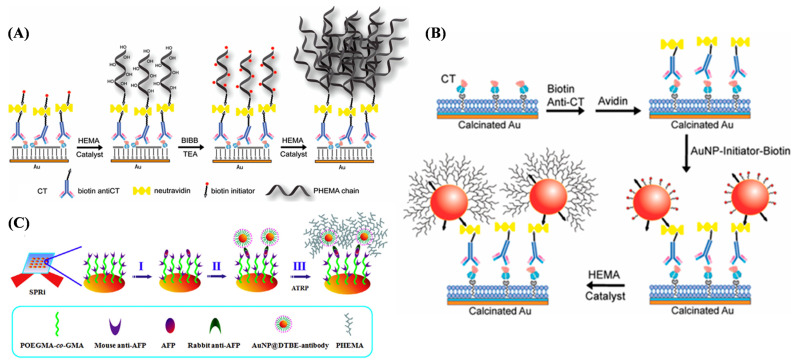
(**A**) Schematic representation of biotinylated initiator-coupled surface and consecutive two steps of in situ surface ATRP reactions for SPR signal amplification [191]. Copyright 2010 American Chemical Society. (**B**) Schematic representation of in situ AuNP coupling through biotin–avidin interaction and surface ATRP reaction for SPR signal amplification in CT detection [192]. Copyright 2012 American Chemical Society. (**C**) Dual SPRi signal amplification [193]. Copyright 2014 Elsevier. Sample was flowed on SPRi chip surface to directly detect AFP target (step I), followed by AuNPs@DTBE–antibody to form immunocomplexes for the first signal amplification (step II), and on-chip ATRP was triggered to further enhance the signal (step III).

The complexity of clinical conditions with various biological matrices may severely influence the reliability and stability of biosensors for direct detection or immersion. Antifouling sensing platforms can effectively reduce undesired binding events to maintain the performance of biosensors. ATRP polymers with antifouling properties have been grafted on sensing interfaces to avoid non-specific binding and enhance target recognition, such as poly[oligo(ethylene glycol) methacrylate-*co*-glycidyl methacrylate] (POEGMA-*co*-GMA), poly(N-isopropylacrylamide) (PNIPAAm), poly(3-acrylamidophenylboronic acid-*co*-2-dimethylaminopropylmethacrylamide, poly-(vinylamine-co-N-vinylformamide), poly[2-methacryloyloxyethylphosphorylcholine (MPC)], and so on [177,178,179,180,181,182,183,184,185,186,187,188,189,190]. More interestingly, ATRP can be in situ-performed by recruiting a large number of monomers at the sensor interface to amplify the optical signal. SPR spectroscopy allows for the label-free, in situ, and real-time monitoring of a broad range of biomolecular interactions in a fast, convenient, and nondestructive way. The adsorption of targets onto the metal surface causes a change in the refractive index near the metal–dielectric interface, which can be measured as a change in the resonance angle. Thus, SPR and SPR imaging (SPRI) techniques have been widely used in the fields of disease diagnosis, drug screening, and food control. However, small molecules and targets with a low refractive index can only lead to a small change, which is difficult to measure. It is one of the most effective signal amplification approaches by increasing the mass of captured targets. Liu et al. proposed an ATRP method for enhancing the SPR signal response based on the in situ growth of polymer brushes of poly(hydroxyl-ethyl methacrylate) (PHEMA) (Figure 22A) [191]. Bacterial cholera toxin (CT) was detected as a model analyte. A biotinylated initiator was attached onto the chip surface covered with biotinylated anti-CT with neutravidin as the linker, triggering the localized ATRP of hydroxyl-ethyl methacrylate (HEMA). When the PHEMA was activated with the additional initiator 2-bromoisobutyrylbromide (BIBB), the second ATRP could be achieved to form hyperbranched polymers, further enhancing the SPR signal. AuNPs acting as tags can further enhance the SPR signal due to the coupling effect between the LSPR of AuNPs and the SPR of the gold chip. Liu et al. reported another signal amplification method to enhance the SPR response in combination with AuNPs with ATRP. A phosphatidylcholine vesicle was coated on the calcinated SPR chip to establish a bilayer membrane for embedding cell receptor monosialoganglioside GM1 (Figure 22B) [192]. After the capture of CT by GM1, biotinylated anti-CT was attached onto the chip surface, allowing for the conjugation of initiator-modified biotinylated AuNPs with avidin as the linker. Then, PHEMA polymer brushes were in situ-formed on the surfaces of AuNPs, further enhancing the SPR signal for quantitative detection of CT at a very low concentration (160 aM). Hu et al. reported an SPR imaging (SPRi) immunoassay platform based on the dual-signal amplification of antibody-modified AuNPs and ATRP (Figure 22C) [193]. The POEGMA-*co*-GMA polymer was grafted onto the chip surface for the immobilization of the captured antibody (anti-AFP). The ATRP initiator bis[2-(20-bromoisobutyryloxy)ethyl]disulfide (DTBE) was modified onto the surface of AuNPs for the immobilization of the recognition antibody through ion pair and hydrogen bond interactions. Capture of AuNPs@DTBE–antibody conjugates by the immunocomplexes on the chip surface achieved the first signal amplification. Then, the initiator triggered on-chip ATRP of HEMA monomers on the surface of AuNPs to further enhance the SPRi signal.

**Figure 23:**
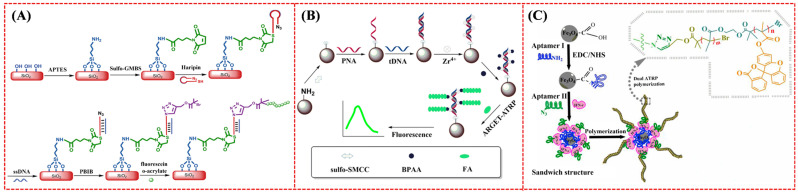
(**A**) Principle of fluorescence detection of sequence-specific DNA via click chemistry and AGET ATRP [199]. Copyright 2019 Elsevier. (**B**) Schematic illustration of fluorescent biological analysis based on ARGET ATRP with EDTA as the metal ligand [194]. Copyright 2020 Elsevier. (**C**) Schematic illustration of ultrasensitive aptamer fluorometric IFN-γ detection by dual ATRP amplification [206]. Copyright 2019 Elsevier.

Fluorescent biosensors are crucial analytical tools for the quantification of various biomarkers and imaging of living cells. Based on Cu(I)-catalyzed alkyne–azide cycloaddition and phosphate–Zr^4+^–carboxylate chemistry, nucleic acids and enzymes have been determined by the formation of fluorescent polymers on different supports based on the signal amplification of ATRP (Table 5) [194,195,196,197,198,199,200]. The polymerization principles are the same as those of the aforementioned electrochemical biosensors, by using fluorescent molecules instead of electroactive monomers. For example, Yang et al. reported a fluorescent method for DNA detection using an N_3_-labeled hairpin DNA probe to capture the target (Figure 23A) [199]. Hybridization of the target DNA with the hairpin probe led to the exposure of the N_3_ group, allowing for the attachment of the AGET ATRP initiator via click chemistry to polymerize numerous fluorescein-o-acrylate (FA) monomers on the silicon surface. Zhang et al. reported a fluorescent method for the detection of CYFRA21-1 DNA based on the signal amplification of ATRP through the bridge of phosphate–Zr^4+^–carboxylate chemistry (Figure 23B) [194]. Plenty of FA monomers were polymerized on the surfaces of MBs to produce a strong fluorescence signal. In addition, through the signal amplification of ATRP in combination with other methods, such as DSN-assisted target cycle, biomacromolecule initiators, and functional nanomaterials, more and more fluorescent molecules can be polymerized on the support surface to enhance the signals [201,202,203].

Among various kinds of biosensors, fluorescent aptasensors have become promising tools for the rapid quantification of antibiotics, drugs, and biomolecules, owing to their significant advantages of simplicity, sensitivity, selectivity, rapid response, and low cost. Fluorescent aptasensors with DNA or RNA strands as the target aptamers have been designed for the detection of OTA, bisphenol A, and proteins in a competitive or sandwich format by ATRP signal amplification [204,205,206,207,208]. The polymers can be pre-prepared and used to label DNA or be in situ-formed for signal readout. For example, Wen et al. designed a sandwich fluorescent aptasensor for the assay of the gamma-interferon (IFN-γ) protein based on the signal amplification of ATRP (Figure 23C) [206]. After the formation of the “aptamer–protein–aptamer” sandwich complex, the ATRP initiator was linked to the N_3_-labeled aptamer by click chemistry, triggering the growth of FA monomers on the nanoparticle surface.

**Figure 24:**
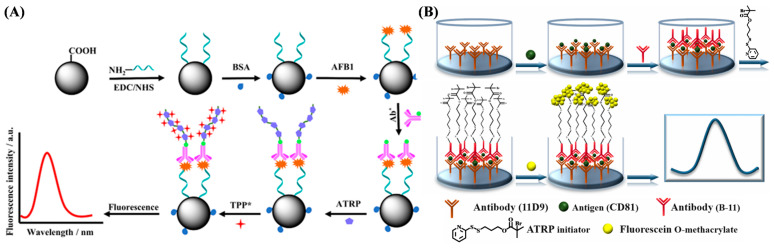
(**A**) Schematic diagram of AFB1 fluorescence sensor based on ARGET-ATRP [210]. Copyright 2022 Elsevier. (**B**) Fabrication process of ELISA sensors prepared by amplifying fluorescence signals via immuno-ATRP assay [211]. Copyright 2024 American Chemical Society.

Fluorescence immunoassays have shown great potential in promoting public health due to their integration of superior specificity in immune recognition and the excellent sensitivity of fluorescence sensors. Polymers have been actively pursued in the construction of fluorescence immunoassays due to their tendency toward surface engineering, high compositional diversity, and structural flexibility. ATRP-based polymers and polymeric materials have been prepared and used for fluorescence immunoassays [29,209]. For example, Guo et al. reported a sandwich-type “aptamer–antigen–antibody” immune system for the detection of aflatoxin B_1_ (AFB_1_) by grafting fluorescent molecules of carboxy porphyrins (TPP*) as signal reporters on the surfaces of magnetic beads (MBs) (Figure 24A) [210]. The antibody was labeled with 2-bromo-2-methylpropionic acid (BMP) to trigger the ATRP of acrylamide monomers. Fluorescent TPP* molecules were then covalently linked to the polymers via amidization. Ma et al. reported a fluorescent immunosensor for the detection of pre-eclampsia protein marker CD81 based on ferritin-mediated ATRP using ELISA kits (Figure 24B). The formed FA polymers with a low molecular weight and uniform chain structure were readily determined with a simple microplate reader [211].

**Figure 25:**
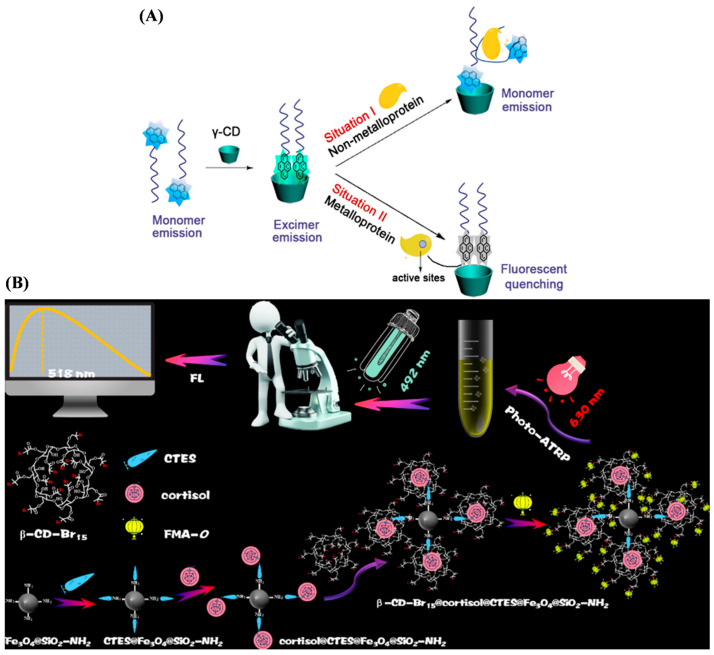
(**A**) Illustration of fluorescent response mechanism of polymer-PyBr/γ-CD inclusion complexes to proteins [212]. Copyright 2016 American Chemical Society. (**B**) Illustration of fluorescent biosensor for cortisol detection [213]. Copyright 2025 Elsevier.

Supramolecular nanotechnology has been successfully used in drug delivery, catalysis, sensing, and other applications. Based on the host–guest chemistry and non-covalent interactions, fluorescent polymers prepared by ATRP reaction have been used for the design of interesting biosensors. The cavities of cyclodextrins (CDs) can serve as appropriately sized guests to capture various hydrophobic molecules due to their internal hydrophobic environment and hydrophilic outer surface. Pyrene-based molecules show the property of fluorescent conversion from monomer emission to excimer emission due to the inter- or intra-aggregation of pyrene monomers. Aqueous pyrene polymers, PDMAEMA and poly(N-acryloyl-glucosamine) (PAGA), have been prepared by using a pyrene-functionalized initiator to trigger ARRP and served as the probes to detect proteins (Figure 25A) [212]. The pyrene tags were accumulated into the cavities of γ-cyclodextrin (γ-CD) to form polymer pyrene/γ-CD complexes, resulting in the appearance of excimer emissions. When binding to non-metalloproteins, the pyrene polymers were released from γ-CD, causing the recovery of monomer emission and the quenching of excimer emission. However, the binding of pyrene polymers with metalloproteins quenched the excimer/monomer emission by energy transfer. The selectivity and sensitivity of this method toward different proteins could be modulated by changing the type and chain length of polymers, providing a promising platform for protein sensing. In addition, CDs are easily derivatized by functional molecules since they have many hydroxyl groups. Wang et al. developed a fluorescent biosensor for cortisol detection using β-CD-Br-_15_ as the guest and ATRP as the initiator. The β-CD-Br_15_ molecule was synthesized by the esterification reaction between β-CD and BIBB (Figure 25B) [213]. Cortisol was captured by the carboxyethylsilanetriol (CTES)-modified Fe_3_O_4_@SiO_2_-NH_2_ nanoparticles through the silanol condensation reaction and then recognized by β-CD-Br_15_ via host–guest chemistry. The anchored β-CD-Br_15_ could initiate the polymerization of fluorescein FMA-O with zinc phthalocyanine (ZnPc) as the photocatalyst under 630 nm radiation.

**Figure 26:**
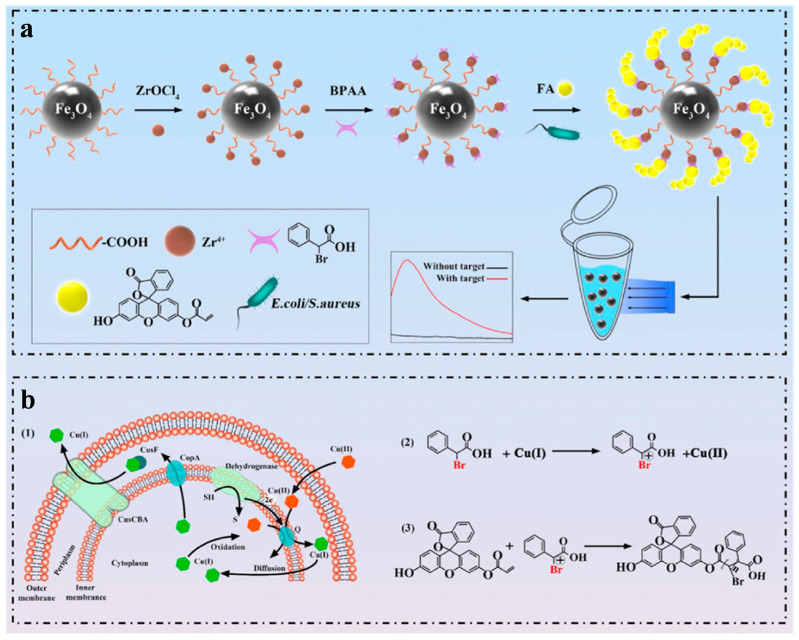
(**a**) Schematic representation of an ultra-sensitive fluorescence biosensor based on bacteria-instructed ATRP polymerization for monitoring foodborne pathogens. (**b**) Schematic representation of bacteria-instructed ATRP progression [214]. Copyright 2024 Elsevier.

In the general ATRP process, an external condition, such as chemical, optical, electrical, and mechanical stimuli, is required to activate the polymerization and growth of polymers by reducing Cu(II) to Cu(I). Pathogenic bacteria show unique Cu(II) binding and reducing capacities. For this view, bacteria-responsive biosensors have been designed through Cu(I)-promoted chemical reactions in combination with appropriate signal amplification techniques. Wang et al. reported a fluorescent biosensor based on bacteria-mediated ATRP for the detection of foodborne pathogenic bacteria [214]. As shown in Figure 26, the initiator of BPAA was modified on the surface of carboxylated Fe_3_O_4_ MBs via the carboxylate–Zr^4+^–carboxylate interaction. Cu(II) was reduced to Cu(I) via the distinctive copper-binding redox pathway of bacteria, activating the polymerization of FA monomers to form fluorescent polymers on the bead surface. The signal was linearly enhanced with the increase in the bacterial (*S. aureus* and *E. coli*) concentration in the range of 10^3^~10^8^ CFU/mL, with a detection limit of 10^2^ CFU/mL.

**Table 5:** Overview on ATRP-based signal amplification for fluorescence biosensors.

Biosensor	Analyte	Signal Amplification	Linear Range	Detection Limit	Ref.
Nucleic acid sensing	DNA	ARGET ATRP	10^−7^–1 nM	23.8 aM	[194]
	DNA	ARGET ATRP	10^−7^–0.1 nM	35.5 aM	[195]
	miRNA-144	ARGET ATRP	10^−3^–100 nM	4.6 fM	[196]
	TMV RNA	ATRP	10^−4^–10 nM	1.14 fM	[197]
	DNA	REase and ATRP	10^−5^–10 nM	0.14 fM	[198]
	DNA	ATRP	10^−7^–1 μM	4.3 fM	[199]
	TMV RNA	DSN and ATRP	10^−2^–100 pM	1.03 fM	[201]
	DNA	Polysaccharide and ATRP	10^−7^–0.1 nM	78 aM	[202]
	DNA	REase and ATRP	10^−5^–10 nM	2.44 fM	[203]
Aptasensor	IFN-γ	Dual ATRP	2 × 10^−9^–5 × 10 nM	1.54 fM	[206]
	OTA	ARGET ATRP	2 × 10^−3^–2 × 10^3^ ng/mL	7.6 fg/mL	[204]
	BPA	ARGET ATRP	10^−4^–100 nM	6.6 fM	[205]
	CEA	β-CD and ATRP	10^−15^–10^−7^ g/mL	6.76 ag/mL	[207]
Immunoassay	AFB_1_, OTA	ARGET ATRP	5–250, 0.5–80 ng/mL	426.18, 79.55 fg/mL	[208]
	Exosome	ARGET ATRP	5 × 10^4^–5 × 10^9^ exosomes/mL	11,610 exosomes/mL	[29]
	CYFRA21-1	UCNPs and ATRP	10^−3^–0.1 ng/mL	38.7 fg/mL	[209]
	CD81	ATRP	10^−4^–10 ng/mL	0.067 pg/mL	[211]

Abbreviations: TMV, tobacco mosaic virus; DSN, duplex-specific nuclease; REase, restriction endonuclease; IFN-γ, gamma-interferon protein; β-CD, β-cyclodextrin; AFB1, aflatoxin B1; OTA, ochratoxin A; UCNPs, upconversion nanoparticles; CD81, pre-eclampsia protein marker.

## 5. Conclusions, Challenges, and Future Perspectives

Sensitive and selective detection is important to determine chemical and biological species. Owing to its capability to precisely regulate the structure and function of polymers, ATRP plays a crucial role in the sensing field by providing core technical support for the design and fabrication of high-performance sensors. This review explored the development and advantages of ATRP-based sensing materials and ATRP-assisted signal amplification. ATRP-based polymers and polymeric materials have abundant sites for the immobilization of specific functional groups and recognition elements for selective recognition of targets. In ATRP-assisted signal amplification strategies, the in-situ formation of polymers at the sensing interfaces can result in the accumulation of a large number of signal probes for direct signal-readout or function groups for further capture of signal probes. Such sensors have demonstrated practical potential in the fields of biomolecule detection, environmental pollutant monitoring, and food safety. However, ATRP still faces urgent challenges to be addressed in sensing applications. First, the residual transition-metal catalysts (even if the dosage is reduced to the ppm level) or ligands from the ATRP process may induce biotoxicity in biomedical sensing scenarios, showing adverse effects on sensor performance and biocompatibility. Second, in complex real samples, some polymer recognition layers prepared via ATRP are prone to non-specific adsorption, thereby impairing the accuracy of detection results. Third, most of the studies are still confined to laboratory conditions and exhibit poor adaptability to on-site detection environments, failing to meet the requirements of practical applications. It is noteworthy that ATRP shows unique advantages and trade-offs in contrast with other RDRP techniques widely used in biosensor development, such as RAFT and nitroxide-mediated polymerization (NMP). For instance, ATRP can offer faster polymerization kinetics and better controllability over polymer chain length. This is advantageous for constructing uniform, dense polymer recognition layers on the sensing interface. However, ATRP relies on transition-metal catalysts, leading to potential biotoxicity issues that are less prominent in metal-free RAFT or NMP systems. RAFT is more compatible with a broader range of monomers (e.g., hydrophilic ones for biological sensing) and can avoid metal residues, but its slower reaction rate may limit the efficiency of the in situ formation of polymers at the sensing interface. Although NMP is very suitable for preparing well-defined polymers since it does not involve the use of metal catalysts, it typically requires higher reaction temperatures, which may compromise the stability of biometric elements (such as antibodies and enzymes) in biosensors. This comparative viewpoint emphasizes that ATRP still has competitiveness in the development of biosensors, especially for scenarios that require fast and precise polymer layer manufacturing. This limitation can be addressed by integrating the advantages of alternative RDRP technologies.

In the future, the development of ATRP polymerization in sensing fields can focus on the following key directions: (1) Both the use of transition-metal catalysts and the presence of other substances in complex biological samples may cause possible interference. It is necessary to introduce anti-nonspecific adsorption units, such as polyethylene glycol, with specific recognition groups to enhance the anti-interference capability and detection efficiency of sensors in complex matrices. (2) On the basis of high sensitivity and selectivity, simultaneous detection of multiple biomarkers is very attractive for bioanalysis. Despite the successful sequential conduction of ATPR and other polymerizations, it is still difficult to precisely and simultaneously control the initiation of different polymerizations at one sensing interface. Thus, more specific ATRP techniques, including initiators and catalysts, should be developed. (3) To date, small molecules with excellent electroactive or optical properties that are accumulated in formed ATRP polymers always serve as the signal molecules. However, other functional molecules can also be integrated into polymers, such as molecular enzyme-like catalysts (e.g., hemin and fluorescein) and electroactive molecules for redox cycling. It is a promising approach to improve the detection performance by coupling ATRP polymerization with other powerful signal amplification strategies. (4) To realize the miniaturization and commercialization of ATRP-based biosensors or instruments, it is necessary to integrate ATRP with emerging platforms for portable on-site sensors, such as paper-based microfluidics, miniaturized electrochemical workstations, and smartphone-based readout systems. In addition, new oxygen-tolerant and copper-free ATRP systems may promote the practical application of ATRP techniques in biosensors. In short, with the continuous deepening of research, ATRP is expected to play a more critical role in the development of various sensors, upgrading their detection performance in environmental monitoring, clinical diagnosis, and food safety, and expanding their application boundaries in practical scenarios.

## Data Availability

No new data were created or analyzed in this study. Data sharing is not applicable to this article.
